# Lignin-derived biodegradable cryogels engineer the rhizosphere to enhance water availability and crop growth

**DOI:** 10.1039/d6ra05329g

**Published:** 2026-11-09

**Authors:** Nisar Uddin, Muhammad Wajid Ullah, Yanwei Xie, Jinxi Zhang, Xin Xie, Daochen Zhu

**Affiliations:** a College of Agriculture, Guizhou University Guiyang 550025 China xxie1@gzu.edu.cn; b International Joint Laboratory on Synthetic Biology and Biomass Biorefinery, Biofuels Institute, School of the Environment and Safety Engineering, Jiangsu University Zhenjiang 212013 China dczhucn@ujs.edu.cn; c Department of Pulp & Paper Engineering, College of Light Industry and Food Engineering, Nanjing Forestry University Nanjing 210037 China

## Abstract

The development of biodegradable soil-conditioning materials that improve water availability without increasing environmental impacts is important for climate-resilient agriculture. Here, we developed lignin-derived cryogels from agricultural waste lignin and evaluate their potential as multifunctional rhizosphere-engineering materials. The optimized formulation (LC_1_) exhibited 2.5-fold greater compressive strength than the lignin-free control and showed enhanced water absorption and sustained water retention at elevated temperatures. LC_1_ also underwent gradual biodegradation, reaching 32.7% mass loss after 130 days of soil burial, while germination indices exceeding 95% indicated negligible phytotoxicity. Agronomic performance was evaluated in three crop species (*Triticum aestivum*, *Zea mays*, and *Brassica napus*) under greenhouse and field conditions. LC_1_ consistently improved crop establishment and growth, with significant increases in plant height, biomass accumulation, chlorophyll content, soluble sugars, and protein levels. These responses coincided with improved root-zone moisture availability associated with the interconnected porous architecture and sustained hydration behavior of the cryogel. Collectively, the results establish experimentally supported structure–property–function relationships linking lignin-dependent modification of cryogel architecture with mechanical resilience, water regulation, biodegradation, and crop performance. This study demonstrates a bio-based strategy for converting underutilized lignin into biodegradable soil-conditioning materials and provides a framework for integrating biomass valorization with sustainable rhizosphere management and climate-resilient agriculture.

## Abstract 2

The development of biodegradable soil-conditioning materials that improve water availability without increasing environmental impacts is important for climate-resilient agriculture.

## Graphical abstract

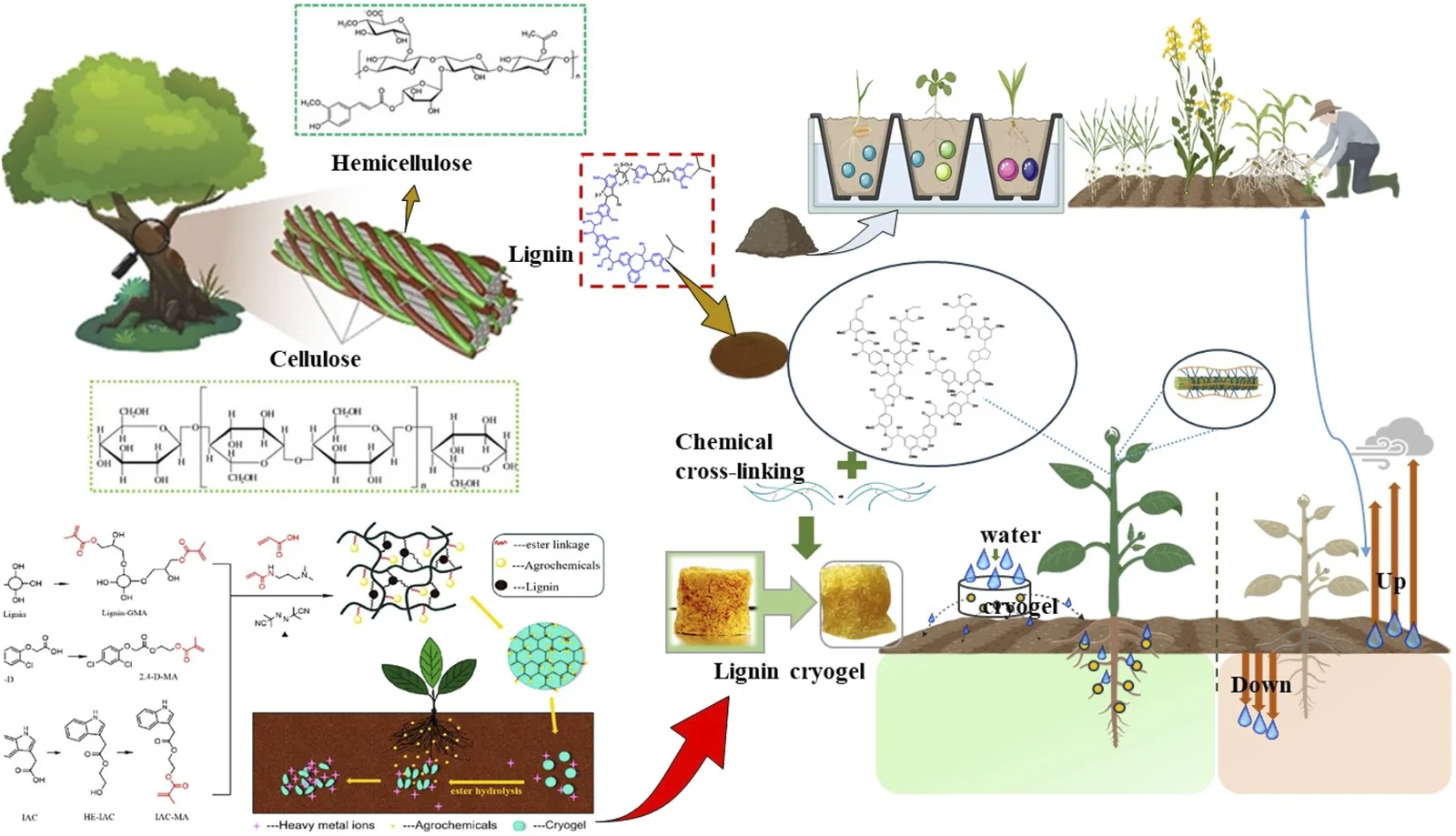

## 1 Introduction

Soil water limitation constitutes a significant constraint to crop establishment and productivity, particularly in nutrient-limited conditions. The global agricultural sector faces increasing pressure from population growth and rising food demand.^1^ According to the United Nations World Population Prospects: The 2017 Revision, the global population was projected to reach approximately 9.8 billion by 2050 and 11.2 billion by 21st century, necessitating an estimated 70% increase in food production^2^ However, conventional agricultural practices, aimed at maximizing yields, often contribute to soil degradation, nutrient leaching, and excessive reliance on agrochemicals, ultimately compromising long-term soil functionality and sustainability.^3–7^ Emphasize the need for resource-efficient soil management strategies that improve water availability during early plant growth while minimizing environmental impacts.

Biopolymers derived from lignocellulosic biomass, particularly lignin, have attracted increasing attention as sustainable materials for agricultural applications.^1,7,8^ Lignin is the second most abundant natural polymer after cellulose and a major structural component of plant cell walls, particularly in woody biomass. Its heterogeneous aromatic structure and abundance of reactive functional groups provide considerable potential for developing biodegradable materials with water-retentive and soil-conditioning properties.^9–11^ Nevertheless, large quantities of industrial lignin remain underutilized and are commonly discarded or combusted for low-value energy recovery, highlighting the need for strategies that convert this abundant biomass resource into value-added agricultural materials.^10,12^ One promising valorization route is the incorporation of lignin into three-dimensional porous polymer networks, such as cryogels, where its chemical functionality can be coupled with structural features favorable for soil-water management. Cryogels are macroporous networks formed under sub-zero conditions and are characterized by interconnected pores, high swelling capacity, and favorable mechanical properties.^13,14^ These characteristics can facilitate water uptake, storage, and gradual release within the soil matrix. Compared with conventional synthetic superabsorbent polymers, lignin-derived cryogels additionally offer biodegradability and reduced environmental persistence, making them attractive candidates for soil amendment in water- and nutrient-limited environments. Previous studies have demonstrated that lignin-based materials can improve soil water retention and modify soil physical properties, with potential benefits for soil functionality and plant performance.^15–18^

Building on these advances, an important question is whether the favorable material properties of lignin-derived cryogels can be translated into effective regulation of the plant rhizosphere. Despite considerable progress in lignin-derived hydrogels and cryogels, previous studies have predominantly focused on material synthesis and physicochemical characterization, while their functional role in regulating rhizosphere processes remains insufficiently understood. In particular, the relationships among cryogel architecture, water-retention dynamics, biodegradation behavior, and plant physiological responses under realistic agricultural conditions have rarely been systematically investigated. Establishing these structure–function relationships is essential for developing next-generation soil-conditioning materials that can simultaneously improve soil moisture management and crop performance. We therefore hypothesized that lignin-derived cryogels can function as biodegradable rhizosphere-engineering platforms by regulating root-zone hydration, sustaining soil moisture availability, and promoting early crop establishment under nutrient-limited conditions.

Therefore, the objectives of this study were to: (i) fabricate and characterize lignin-based cryogels with tunable structural and physicochemical properties; (ii) evaluate their mechanical stability, water-retention behavior, and biodegradation characteristics; and (iii) establish mechanistic relationships between cryogel structure and plant growth responses under greenhouse and field environments. By integrating biomass valorization with rhizosphere engineering, this work provides a sustainable strategy for transforming underutilized lignin resources into functional agricultural biomaterials for climate-resilient crop production.

## 2 Materials and methods

### 2.1 Materials

The materials used in this study included lignin keratin powder, gum arabic (degree of deacetylation exceeding > 90%; Sigma CAS no. 9012-76-4). Other chemicals included glycerol (Macklin, CAS No. 56-81-5), *N*-vinylpyrrolidone (Aladdin, CAS No. 88-12-0), tetramethylethylenediamine (TEMED, Sigma, CAS no. 110-18-9), *N*,*N*′-methylenebisacrylamide (MBA; Sigma, CAS no. 110-26-9), and ammonium persulfate (APS, Sigma, CAS no. 7727-54-0). These reagents were chosen for their well-established roles in polymerization, cross-linking, and structural stabilization, which are crucial for the effective synthesis and functionalization of cryogels.

### 2.2 Lignin extraction from agricultural waste

Lignin was extracted from agricultural waste derived from plant debris, including stems and leaves. The plant materials were meticulously washed to remove impurities, combined, and oven-dried at 50 ± 5 °C for 10 days. After drying, the samples were ground into a fine powder and. Lignin was then extracted in two stages: first with a methanol–water mixture (2 : 1, v/v), followed by water extraction to isolate different lignin fractions. The lignin composition was quantified using a modified thioacidolysis method,^19^ with 4,4-ethylidene bisphenols as the internal standard. The total lignin content was determined by summing the yields of all thioacidolysis-derived monomers.

### 2.3 Synthesis of lignin-based cryogels

Lignin-based cryogels were prepared using lignin, keratin (K) powder, glycerol (Gly), gum arabic (GA), *N*-vinylpyrrolidone (V), tetramethylethylenediamine (TEMED), ammonium persulfate (APS), and *N*,*N*′-methylenebisacrylamide (MBAm). Cryogelation was performed by low-temperature polymerization at −20 °C. Initially, 0.12 g each of GA and keratin powder were dispersed in 20 mL of double-distilled water. Subsequently, 0.16 g MBAm was added as the cross-linking agent, followed by 500 µL of *N*-vinylpyrrolidone. After thorough homogenization, 1 mL glycerol was added, followed by 2 mL of 2 wt% APS solution and 200 µL TEMED to the pre-cooled reaction mixture. The mixture was vigorously stirred in an ice bath, transferred into cylindrical molds (10–12 mm diameter), and maintained at −20 °C for 18–24 h to complete cryopolymerization. The frozen cryogels were then thawed at room temperature for 24 h. The lignin-free formulation was designated NC_0_ (non-lignin control cryogel). Using the same synthesis procedure, lignin was incorporated at two concentrations: 0.02 g for LC_02_ (lignin-containing cryogel, 0.02 g lignin) and 0.10 g for LC_1_ (lignin-containing cryogel, 0.10 g lignin). Following synthesis, all cryogels were purified by immersion in deionized water for 24–36 h, with the water replaced every 4 h to remove residual or unreacted components. The purified cryogels were subsequently freeze-dried and stored in a desiccator until further characterization and experimental use (Fig. 1).

**Fig. 1:**
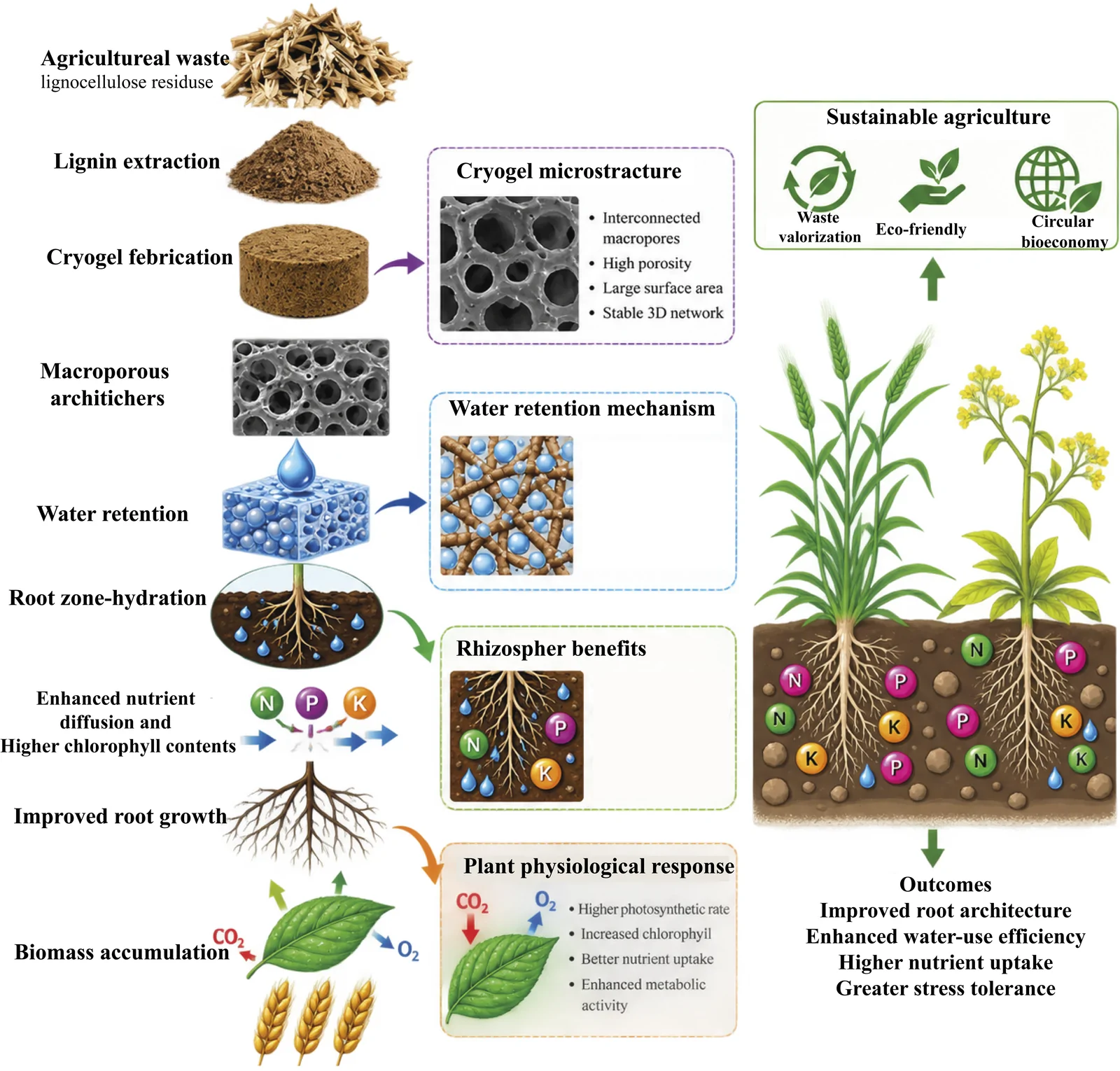
Schematic representation of lignin-based cryogel development and multifunctional applications. Lignocellulosic biomass is processed into solubilized lignin, crosslinked, and cryo-templated to form porous cryogels with tunable morphology and surface functionality, enabling uses in sustainable agriculture.

### 2.4 Characterization of lignin-based cryogels

The morphology of the cryogels was examined using field-emission scanning electron microscopy (FE-SEM, JSM-700IF, JEOL, Japan). A thin gold layer was sprayed to the cryogel surface to enhance imaging resolution.^20^ Cryogel porosity was assessed using mercury intrusion porosimetry (Poremastier-60 Quantachrome, USA). Mercury infiltration was conducted at increasing pressures, with the Washburn formula (eqn (1)) used to correlate pore size with pressure:1
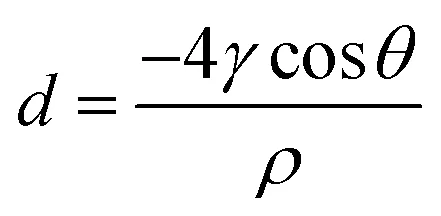
where ‘*d*’ is the pore diameter, ‘*ρ*’ is the pressure, ‘*γ*’ is the surface tension of mercury, and ‘*θ*’ is the contact angle. The pressure was maintained below 40 psi (0.28 MPa) to prevent compression and was increased to 60 000 psi (413.7 MPa) for specimens with permanent porosity.^20^

Fourier transform infrared spectroscopy (FTIR) was performed using a Nicolet iS50 spectrometer (Thermo Electron, USA) to analyze the functional groups present in the cryogels. A total of 16 scans were conducted with a resolution of 4 cm^−1^. Thermal stability was evaluated using a thermal analyzer (NETZSCH TG 209 F3, Germany), where samples were heated from 30 °C to 800 °C at a 20 mL min^−1^ N_2_ flow, maintaining a pressure of 0.3 Pa and a heating rate of 10 °C min^−1^. Differential scanning calorimetry (DSC) measurements were performed using a DSC 4000 instrument (PerkinElmer, USA), monitoring the heat flow from 0 °C to 200 °C at a 20 mL min^−1^ N_2_ flow rate, 0.3 Pa pressure, and a heating rate of 20 °C min^−1^.

### 2.5 Soil, plant materials, and experimental setup

A seed germination experiment was conducted in a greenhouse under controlled temperature conditions. The experimental soil was collected from the 0–20 cm surface layer in Guiyang, Guizhou Province, China. Based on the regional soil classification commonly used in Guizhou, the soil was identified as yellow soil. Because diagnostic profile-level characteristics required for formal classification under USDA Soil Taxonomy or the World Reference Base for Soil Resources (WRB) were not determined, no international taxonomic class was assigned.^21^ The soil had a clay loam texture, a pH of 5.2, and an electrical conductivity of 1.6 dS m^−1^ (1 : 5 soil–water suspension, 25 °C). Three treatments (NC_0_, LC_02_, and LC_1_) were applied, each with three replicates. Each treatment consisted of three independent replicate pots (*n* = 3), with seven seeds planted per pot for each of the three crop species: *Triticum aestivum* L. (WT), *Zea mays* L. (MZ), and *Brassica napus* L. (BR). Individual seedlings within each pot were considered subsamples, and their measurements were averaged to obtain a single biological replicate value for each pot. Accordingly, the pot was considered the experimental unit (*n* = 3 per treatment per species) for all statistical analyses. The growth conditions were maintained at 19 °C during the light period and 17 °C during the dark period, with a light intensity of 16 000 lux and an 8 h photoperiod for 21 days. Treatments were applied every 5 days. The untreated control refers to plants grown without cryogel amendment. The designations CnMZ, CnWT, and CnRP refer to the corresponding comparative control groups for maize (MZ), wheat (WT), and rapeseed (RP), respectively.

A completely randomized block (CRBD) was used, with four treatments (control, NC_0_, LC_02_, and LC_1_) and four replicates per treatment. The experimental plot size was 2 m × 2 m, with seeds sown at recommended densities. Treatments (5 ppm; 200 mL per row) were applied every 5 days. After 22 days, growth traits were recorded, including germination rate, plant height, leaf number and area, root length and biomass, shoot biomass, and total plant biomass.

### 2.6 Water-uptake capacity of cryogel

To assess water uptake in soil, 0.2 g of each cryogel formulation was mixed with a predetermined amount of dry soil and transferred to plastic cups, with soil without cryogel serving as the control. Each cup received 100 mL of water. The initial dry mass (*W*_0_) and hydrated mass at each measurement time (*W*_*t*_) were recorded until equilibrium was reached. Water uptake (*W*_p_) was expressed as the percentage increase in mass relative to the initial dry mass according to eqn (2):2
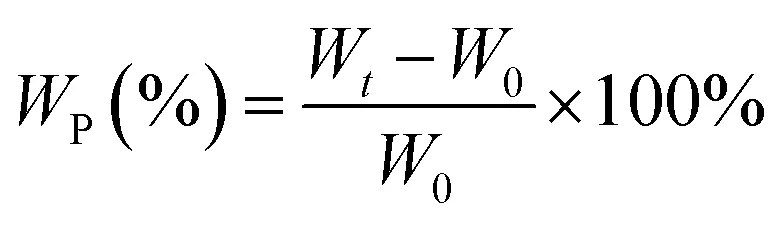
where *W*_0_ is the initial dry mass and *W*_*t*_ is the mass after water absorption at time *t*.

Water-retention behavior was evaluated separately by incubating hydrated cryogels at 35, 45, 65, and 75 °C at 50% relative humidity. The mass remaining during drying was expressed as the relative mass retained *M*_r_.3
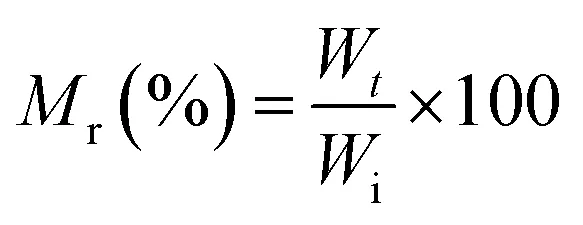
where *M*_i_ is the initial hydrated mass before drying and, *W*_*t*_ is the mass remaining at time *t*.

### 2.7 Soil water retention and hydration dynamics

Soil water-holding capacity (WHC) was determined using the gravimetric saturation method. For each treatment, 100 g of air-dried soil was saturated with distilled water and allowed to drain under gravity for 24 hours. The wet weight (*W*_wet_) was recorded, and samples were subsequently oven-dried at 105 °C for 24 hours to obtain dry weight (*W*_dry_). WHC (%) was calculated using eqn (4):4
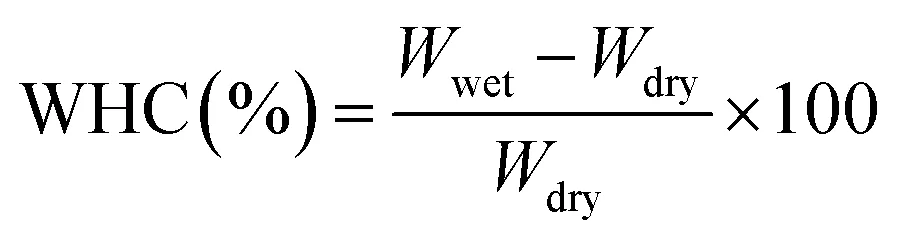
where *W*_wet_ represents the mass of soil after saturation and gravitational drainage for 24 h, and *W*_dry_ represents the mass of the same soil sample after oven-drying at 105 °C for 24 h. Soil moisture retention was monitored over 72 hours utilizing a digital soil moisture probe, supplemented with gravimetric validation on subsamples. Moisture decay curves were constructed to compare water loss across treatments.

### 2.8 Leaf physiological and biochemical measurements

Total chlorophyll concentration was determined using Arnon's method. Briefly, 20 mg of intact, fully developed leaves from wheat, maize, and rapeseed were collected on day 5, 10, 15, and 21. The seedlings were then frozen and ground into a fine powder using a test tube containing steel balls and liquid nitrogen (JXFSTPRP-CL-BSC Freeze Mill, Shanghai Jingxin, China). An extraction mixture was prepared by combining ethanol, acetone, and distilled water in a 4.5 : 4.5 : 1 volumetric ratio. Next, 1.8 mL of this mixture was added to each test tube containing the leaf powder, and the mixture was shaken thoroughly. The samples were centrifuged at 8000 rpm for 1 min to separate the solid particulates from the supernatant. After centrifugation, 1 mL of the clear supernatant was collected for chlorophyll analysis. The absorbance of chlorophyll-a and chlorophyll-b was measured at 645 nm and 663 nm, respectively, using a UV spectrophotometer (Multiskan SkyHigh full-wavelength enzyme marker). The total chlorophyll concentration and soluble sugar in each sample were then calculated using eqn (5) and (6).^22^5
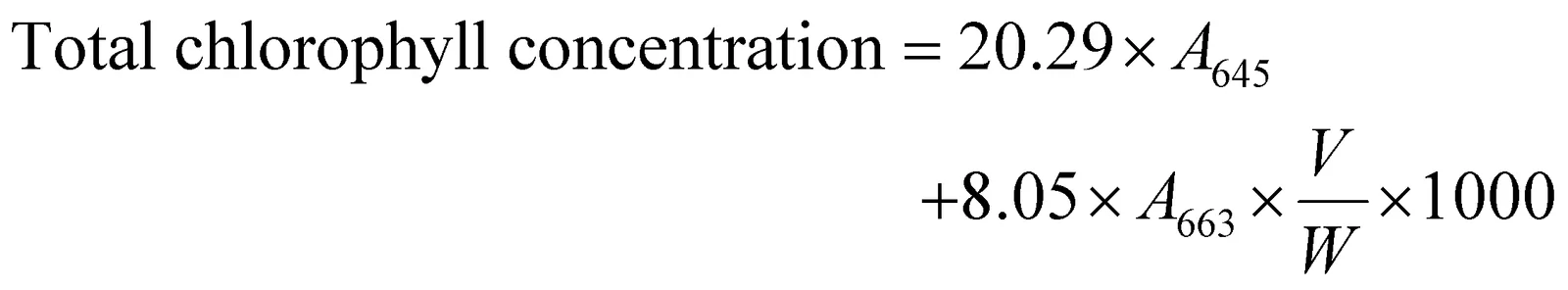
6
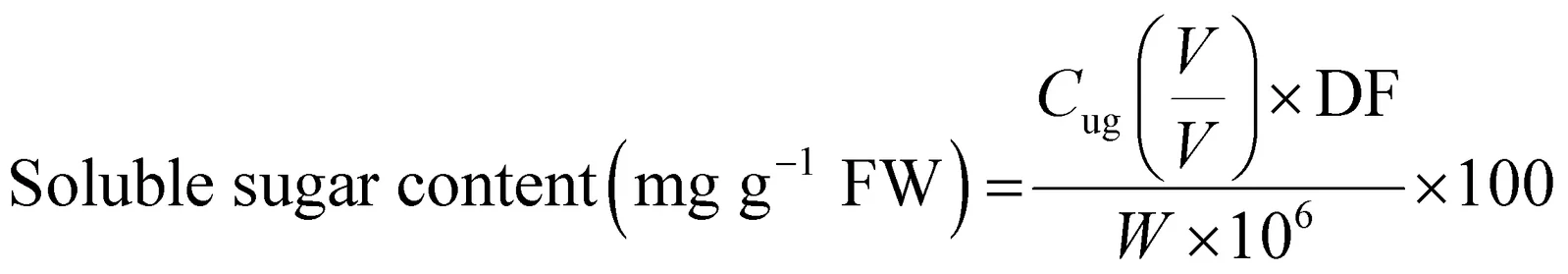
where *C*_ug_ is the soluble sugar concentration determined from the standard curve (µg mL^−1^), (*V*) is the total volume of the extract (mL) DF is the dilution factor, and *W* is the fresh weight of the plant sample (g).

A total of 0.10 g of fresh shoot sample was homogenized with 5 cm^3^ of pre-chilled 10% (m/v) trichloroacetic acid in a 100% acetone solution containing 0.07% β-mercaptoethanol, using a chilled pestle and mortar. The homogenate was kept at −20 °C for 2 hours for protein precipitation, followed by centrifugation at 10 000 g and 4 °C for 15 minutes. The supernatant was discarded, and the pellets were washed with 100% pre-chilled acetone and centrifuged under the same conditions to isolate the protein pellet. This washing was repeated three times. After removing the supernatant, the pellet was dissolved in 1 M NaOH. The protein concentration was quantified by measuring absorbance at 660 nm,^23^ using bovine serum albumin as the standard.

### 2.9 Biodegradation of lignin-based cryogels

The biodegradation of lignin-based cryogels was assessed using the soil burial method, making the first investigation into the degradation potential of lignin-derived cryogels using soil conditions. Cryogels were encapsulated in 60-mesh nylon bags and buried at depths of 4 to 7 cm in the soil. To maintain uniform experimental conditions, soil moisture was maintained at 45% of field capacity throughout the environment. Three biological replicates were used to enhance the reliability of the results and extend the observation period for cryogel degradation. At 15 days intervals, the cryogel samples were retrieved, carefully rinsed to remove adhering soil particles, and then dried and weighed. The degradation rate was calculated for each time point based on the observed change in mass. To improve accuracy, an equivalent quantity of unburied cryogels was processed under identical filtration conditions, ensuring standardization and minimizing potential experimental errors related to handling or measurement variability.

### 2.10 Phytotoxicity bioassay

The phytotoxicity of the cryogel formulation was evaluated using WT, MZ, and RP seeds. Prior to treatment, the seeds were surface-sterilized using a diluted hydrogen peroxide (H_2_O_2_) solution, following the previously reported method,^24^ to eliminate microbial contaminants and ensure uniform conditions for germination. For each treatment, 15 WT seeds were placed in sterile Petri dishes containing either distilled water (control) or cryogel samples saturated with distilled water. The Petri dishes were incubated in complete darkness at 25 °C for 72 h in a controlled growth chamber to promote uniform germination. Phytotoxicity was quantified using the germination index (GI), a widely recognized metric that considers both the percentage of germinated seeds and the mean root elongation. The GI was calculated using eqn (7):7
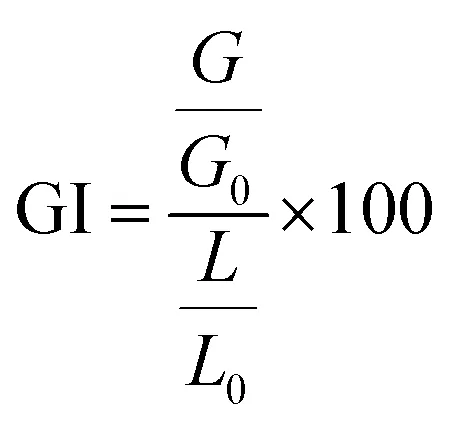
where ‘*G*’ and ‘*G*_0_’ represent the number of germinated seeds in treatment group and control group (double distilled water), respectively, and ‘*L*’ and ‘*L*_0_’ represent the average root length in the treatment group and control group, respectively. A GI value greater than 80% indicates no phytotoxicity, values between 50% and 80% suggest moderate phytotoxicity, and values below 50% indicate high phytotoxicity. This dual-parameter assessment offers a comprehensive evaluation of the impact of cryogel treatments on early seedling development.

A conventional polyacrylamide (PAAm) hydrogel was used as the synthetic hydrogel control (St) for comparison in the germination assay. The hydrogel was prepared by free-radical polymerization of acrylamide (AAm), with *N*,*N*′-methylenebisacrylamide (MBAm) as the crosslinking agent and ammonium persulfate (APS)/tetramethylethylenediamine (TEMED) as the redox initiation system. Briefly, AAm and MBAm were dissolved in deionized water to obtain a homogeneous precursor solution. After degassing to minimize oxygen-mediated inhibition of polymerization, freshly prepared APS was added, followed by TEMED. The mixture was immediately transferred into molds and allowed to polymerize at room temperature until complete gel formation. The resulting hydrogel was thoroughly washed with deionized water, with repeated water replacement to remove residual monomers and initiator, and subsequently freeze-dried before use. The resulting material was designated St and evaluated under the same germination conditions as the cryogel treatments.

### 2.11 Statistical analysis

All quantitative data were expressed as mean ± standard error (SE). For the greenhouse experiments, each treatment comprised three independent replicate pots (*n* = 3) for each plant species, with individual seedlings within each pot treated as subsamples and their measurements averaged to obtain a single replicate value. Differences among treatment groups within individual experiments were evaluated using one-way analysis of variance (ANOVA) followed by Tukey's honestly significant difference (HSD) test for multiple comparisons. For variables evaluated under both greenhouse and field conditions, two-way ANOVA were performed with treatment and environment (greenhouse *vs.* field) as fixed factors to determine the main effects of treatment and environment and their interaction (treatment × environment). 95% confidence intervals were calculated where appropriate. Statistical analysis and graphical visualizations were performed using GraphPad Prism (version 8.0.2, San Diego, CA, USA). Differences were considered statistically significant at *P* < 0.05.

## 3 Results and discussion

### 3.1 Engineering porous lignin cryogels with enhanced structural integrity

The development of biodegradable soil-conditioning materials requires the integration of structural stability, water-management capability, and environmental compatibility. In this study, agricultural waste-derived lignin was incorporated into a keratin-based cryogel matrix to generate highly porous three-dimensional architectures suitable for rhizosphere engineering applications (Fig. 2). SEM imaging revealed clear lignin-dependent changes in cryogel morphology. The lignin-free NC_0_ formulation exhibited a comparatively compact and heterogeneous structure with less-developed pore connectivity, whereas, LC_02_ and LC_1_ showed more open, interconnected macroporous network continuous channels, and thinner pore walls (Fig. 2e–g). Quantitative analysis further demonstrated that the mean pore diameter increased from 54.18 ± 17.25 µm in NC_0_ (Fig. 2e), to 80.04 ± 28.68 µm in LC_02_ (Fig. 2f), and 95.87 ± 34.43 µm in LC_1_ (Fig. 2g). The corresponding mean pore-wall thicknesses were 13.16 ± 3.50, 14.30 ± 7.13, and 18.42 ± 5.51 µm (Fig. 2e–g). Importantly, the 50, 100, and 200 µm scale bars displayed in the SEM micrographs indicate only the spatial scale of the respective images and are distinct from the quantitatively determined pore diameters and pore-wall thicknesses. These measurements indicate that increasing lignin incorporation was associated with enlargement of the macroporous architecture and increased pore-wall thickness. Such structural changes may influence water uptake, storage, and transport within the cryogel network. Previous studies have similarly associated lignin incorporation with the development of porous cryogel structures and enhanced water-retention performance.^25,26^

**Fig. 2:**
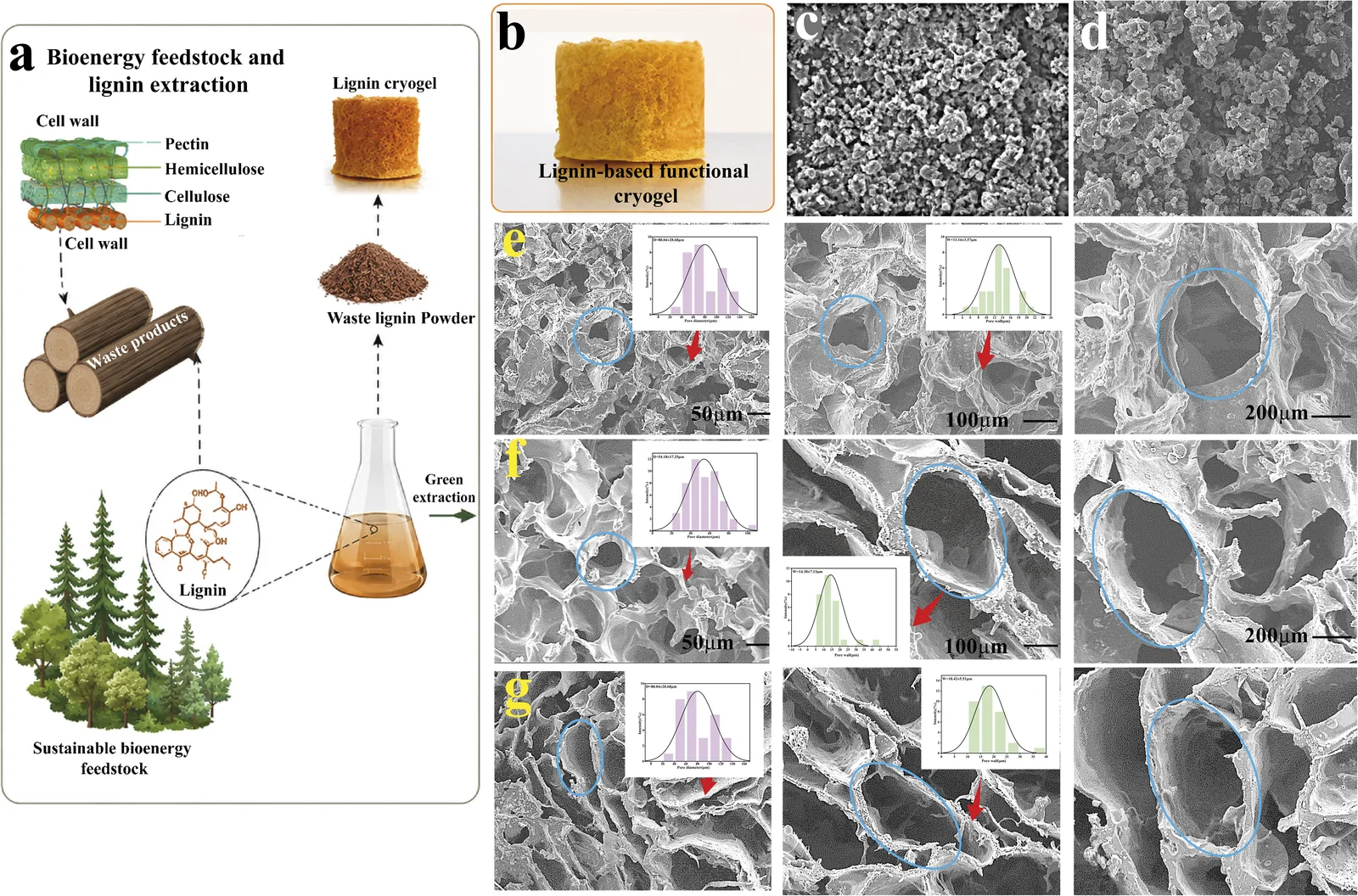
(a) Schematic illustration of bioenergy feedstock and lignin extraction from plant biomass. (b) Lignin-based functional cryogel. (c and d) SEM micrographs of lignin and keratin control matrix. (e–g) SEM micrographs of lignin-containing cryogel showing hierarchical macroporous structures in NC_0_, LC_02_ and LC_1_ formulations. Scale bars represented 50 µm, 100 µm, and 200 µm.Lignin-mediated modification of cryogel physicochemical properties.

The structural differences among NC_0_, LC_02_, and LC_1_ further indicate that lignin incorporation influenced network formation during cryogelation. During freezing, lignin-mediated changes in phase separation and ice-crystal formation may contribute to the generation of larger interconnected voids that remain after thawing. The ∼2.5-fold increase in compressive strength following lignin incorporation suggests reinforcement of the cryogel network through enhanced cross-linking and intermolecular interactions between lignin and the polymer matrix. Such interactions can strengthen molecular connectivity within gel networks, improve stress transfer, and consequently enhance mechanical stability, consistent with recent reports demonstrating that reconstruction and reinforcement of intermolecular networks markedly improve the mechanical robustness of polymeric gels.^27–29^ From a functional perspective, the interconnected macroporous architecture provides pathways for water uptake and retention within the cryogel network and may facilitate subsequent water release during drying. The open structure may also support gas exchange within the surrounding soil matrix.

The physicochemical characteristics of lignin-derived cryogels play a critical role in determining their hydration behavior, biodegradability, mechanical resilience, and overall suitability for rhizosphere engineering applications. To elucidate the structural effects of lignin incorporation, Fourier-transform infrared spectroscopy (FTIR), differential scanning calorimetry (DSC), thermogravimetric analysis (TGA), and X-ray diffraction (XRD) analyses were performed to investigate the chemical interactions, thermal behavior, and structural organization of the cryogel network (Fig. 3).

**Fig. 3:**
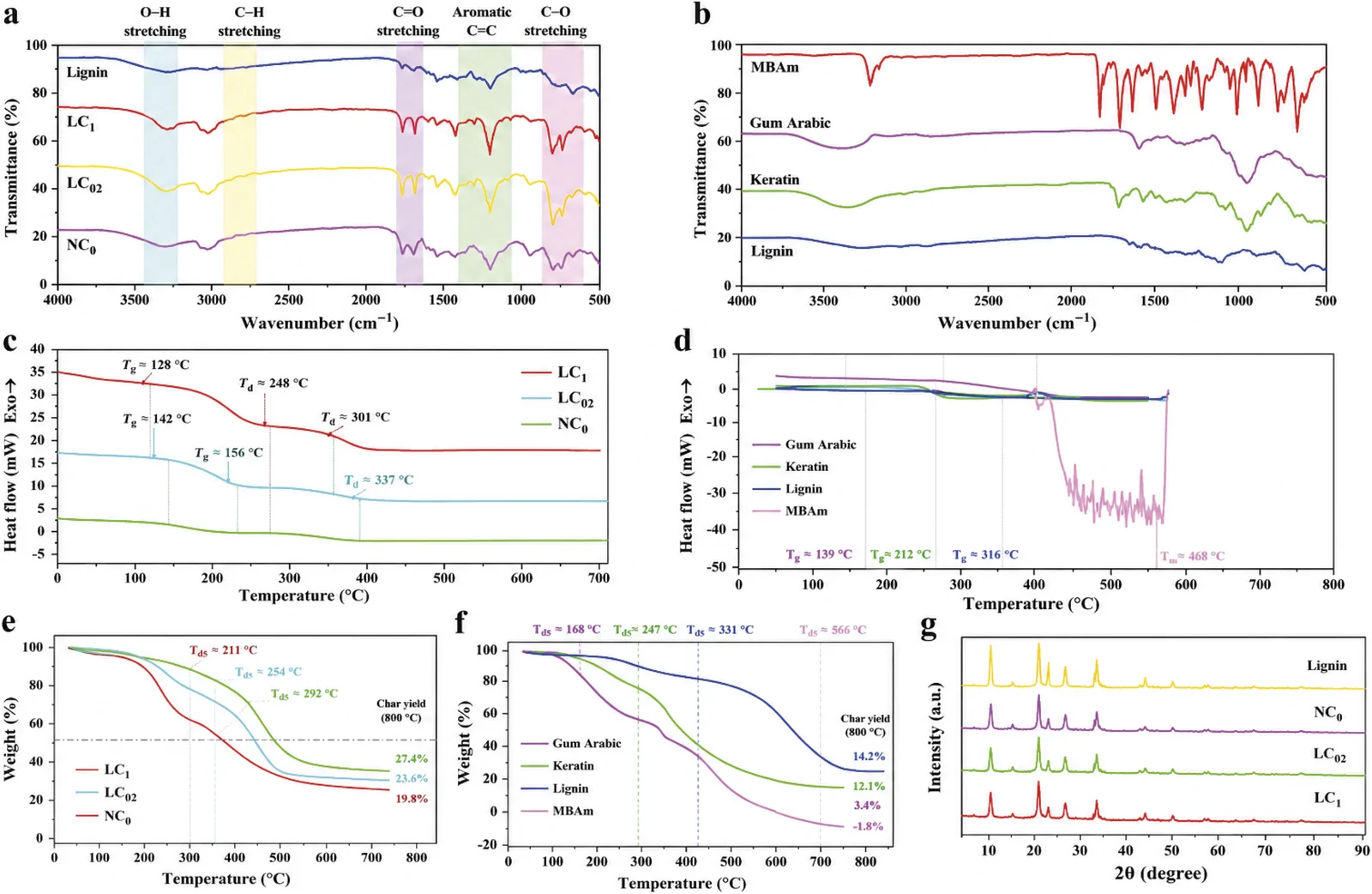
Physicochemical characterization of lignin-based cryogel (a and b) physicochemical characteristics support the functional suitability of lignin-based cryogels as biodegradable materials with improved stability and moisture–interaction properties relevant to soil composite cryogel (NC_0_, LC_02_ and LC_1_), and individual components including MBAm, gum arabic, keratin, and extracted lignin. (c and d) DSC thermograms of cryogel formulations. (e and f) TGA of cryogel composite and individual components. (g) XRD patterns of lignin and cryogel formulations.

FTIR analysis confirmed the characteristic functional groups of the individual components and their retention with in the lignin and lignin-based cryogel (Fig. 3a and b and Table S1). All lignin-based cryogels exhibited a broad absorption band at 3276–3280 cm^−1^ corresponding to N–H and O–H stretching vibrations, associated with lignin hydroxyl groups and the keratin and lignin hydroxyl groups. Notably, this band in LC_02_ and LC_1_ was consistent with increased intermolecular hydrogen-bonding interactions following lignin incorporation. The band near 2930 cm^−1^ was assigned primarily to aliphatic C–H stretching vibrations.^30,31^ Additional spectral features in the ∼1600–1700 cm^−1^ region were associated predominantly with carbonyl/amide I vibrations, whereas bands in the ∼1500–1600 cm^−1^ region included aromatic skeletal vibrations of lignin together with contributions from the proteinaceous matrix. Absorption features within the ∼1000–1300 cm^−1^ region were attributed to C–O stretching of lignin-derived hydroxyl/ether functionalities and contributions from amide III vibrations of keratin. The persistence of these characteristic bands in LC_02_ and LC_1_ supports the incorporation of lignin while retaining the principal chemical features of the keratin-based cryogel network. The major FTIR bands and their assignments are summarized in Table S1. These spectral features demonstrate the formation of a structurally integrated lignin–keratin composite network capable of combining the hydrophilic nature of keratin with the aromatic functionality of lignin.^32^ From a functional perspective, these molecular interactions are highly relevant for agricultural applications. The coexistence of hydrophilic functional groups and aromatic domains creates a multifunctional network capable of simultaneously adsorbing water, maintaining structural integrity, and resisting rapid degradation. Such characteristics are advantageous for maintaining sustained moisture availability within the rhizosphere while preserving material functionality during critical stages of crop establishment.^25,33^

Differential scanning calorimetry (DSC) analysis revealed different changes in the thermal transition behaviour of the cryogel formulation following lignin incorporation (Fig. 3c and d). LC_1_ exhibited a glass-transition temperature (*T*_g_) of 128 °C, followed by thermal transitions at *T*_d1_ = 248 °C and *T*_d2_ = 301 °C, whereas LC_02_ exhibited *T*_g_ = 142 °C, *T*_d1_ = 156 °C, and *T*_d2_ = 337 °C (Fig. 3c). These differences demonstrate that lignin incorporation modified the thermal behavior of the cryogel network. Analysis of the individual components further showed characteristic transitions at approximately 139 °C for gum arabic, 215 °C for keratin, 316 °C for lignin, and 468 °C for MBAm (Fig. 3d). These shifts in thermal transitions between the individual components and composite cryogels are consistent with changes in intermolecular interactions and polymer-chain organization within the cryogel matrix.^34,35^

Thermogravimetric analysis (TGA) further demonstrated composition-dependent difference in thermal degradation (Fig. 3e and f). The temperature corresponding to 5% mass loss (*T*_5_%) was 211 °C for LC_1_, 254 °C for LC_02_, and 292 °C for NC_0_ (Fig. 3e). At 800 °C, the corresponding residual char yields were 19.8%, 21.6%, and 27.4%, respectively. Analysis of the individual components showed *T*_5_% values of 164 °C for gum arabic, 247 °C for keratin, 331 °C for lignin, and 361 °C for MBAm, with residual char yields at 800 °C of 4.0%, 12.1%, 14.2%, and 1.8%, respectively (Fig. 3f). These quantitive differences confirm that incorporation of lignin altered the thermal–decomposition profile of the composite network. The relatively high *T*_5_% isolated lignin and its residual char formation are consistent with the contribution of its aromatic structure to thermal behavior.^35,36^

X-ray diffraction (XRD) analysis was used to examine structural organization within the cryogel formulations (Fig. 3g). Isolated lignin exhibited a broad diffraction feature, consistent with limited long-range molecular ordering. In comparison, NC_0_ displayed several relatively distinct diffraction peaks, while the diffraction profiles of LC_02_ and LC_1_ showed changes in peak intensity and broadening following lignin incorporation. Because peak broadening alone cannot provide a quantitative measure of crystallinity or amorphous content, these changes are interpreted conservatively as evidence that lignin incorporation altered molecular packing and structural ordering within the cryogel matrix rather than as definitive proof of increased amorphous content. Quantitative changes in crystallinity were avoided in the absence of a crystallinity-index analysis. FTIR, DSC, TGA, and XRD analyses demonstrate that incorporating lignin fundamentally modifies the cryogel network's chemical environment, structural organization, and thermal stability. Extensive hydrogen bonding, increased amorphous content, and enhanced thermal resistance yield a multifunctional material that balances hydration, stability, and biodegradability. These characteristics provide the mechanistic basis for the improved water retention, controlled degradation, and plant-growth-promoting effects observed in lignin-derived cryogels throughout this study. More importantly, these findings establish a direct structure–property relationship, linking molecular interactions within the gel network to its functional performance in rhizosphere engineering applications.

### 3.2 Water regulation and biodegradation behavior of lignin cryogels

The stability, biodegradation characteristics, and water management performance of lignin-based cryogels were systematically evaluated to determine their functional suitability as sustainable soil-conditioning biomaterials. Traditional synthetic hydrogels, particularly those based on acrylic acid and polyacrylamide, have been widely employed to enhance soil water retention. However, these materials exhibit low biodegradability and possess a prolonged lifespan within the soil ecosystem, leading to significant environmental and ecological concerns.^37–39^

In contrast, cryogels synthesized from lignin, konjac glucomannan, and sodium alginate exhibited tunable mechanical strength and controllable biodegradability, highlighting the inherent susceptibility of lignin-rich scaffolds to microbial degradation.^40^ Analysis indicated that the incorporation of lignin significantly enhanced the compressive strength and elastic recovery of cryogels (Fig. 4a and b). The LC_1_ variant, subjected to 80% compressive strain, exhibited a maximum stress resistance of ∼450 kPa, whereas the NC_0_ cryogels demonstrated a maximum stress resistance ∼200 kPa. This increase in compressive strength, approximately 2.5-fold, serves as a direct indication that lignin acts as an effective structural reinforcement material, improving network cross-linking density and load-bearing capacity, which are critical for maintaining pore integrity during soil compression. Notably, the complete recovery of LC_1_ following compression demonstrates its elastic strength, which is essential for sustaining soil structural conditioning during cycles of repeated wetting and drying. Such mechanical stability is vital for preserving rhizosphere pore connectivity, thereby facilitating ongoing water diffusion and root penetration, which underpins the stimulated root elongation and biomass accumulation observed in lignin cryogel-treated plants (Fig. 4).

**Fig. 4:**
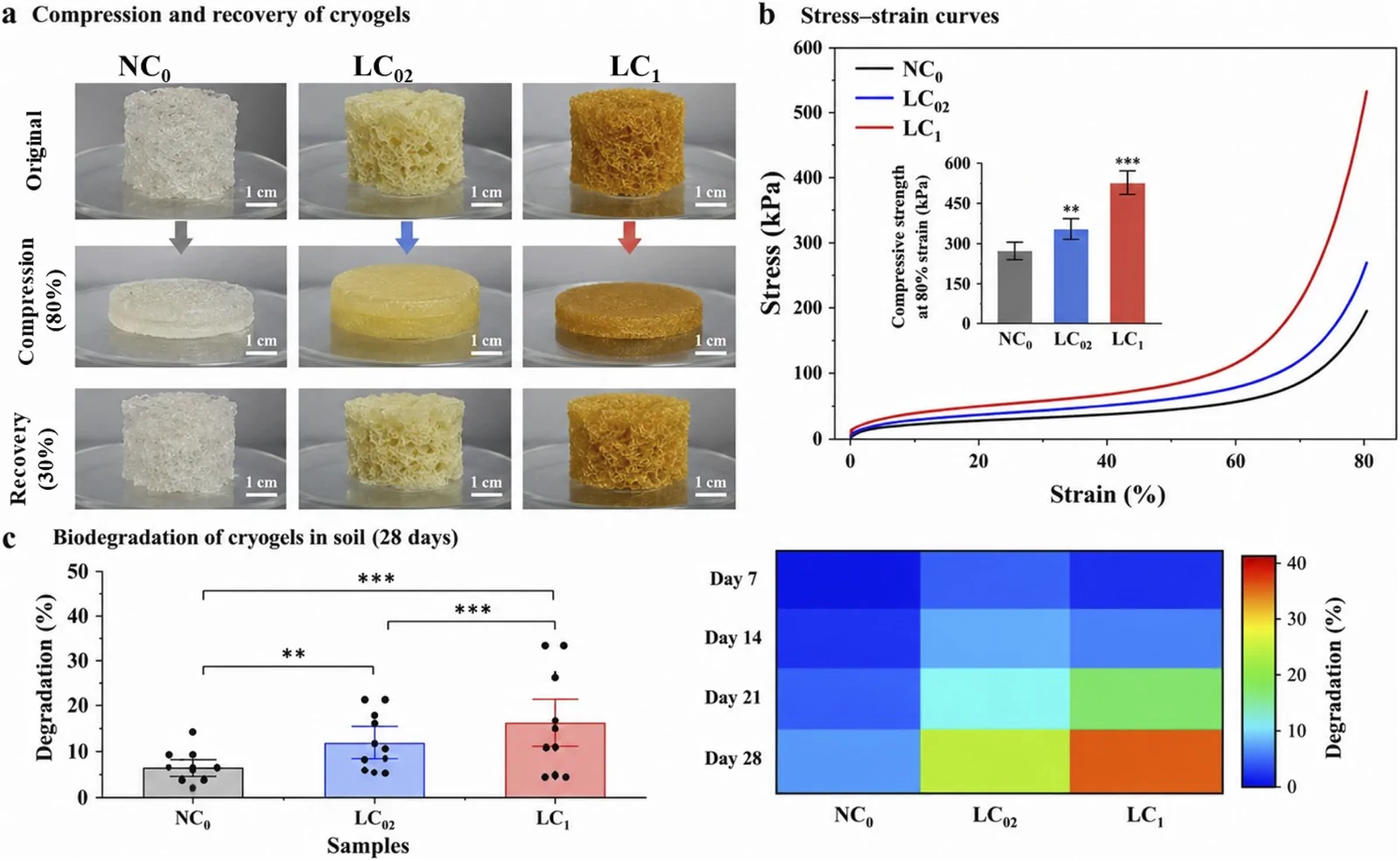
Mechanical resilience and biodegradation of lignin. (a) Cryogel under compression and recovery demonstrate structural resilience. (b) Stress–strain curves show enhanced strength with lignin incorporation (LC_02_, LC_1_) compared with NC_0_. (c) The biodegradation profile displayed as bar plots and a heatmap reveals higher degradation in the lignin cryogel. Early-phase biodegradation profile of the lignin-based cryogels during the first 28 days (0–28 days) of the full 130 days soil-burial degradation experiment. The complete degradation study was continued for 130 days. Data are mean ± SD; **P < 0.01, ***P < 0.001 (one-way ANOVA).

The analysis of biodegradation revealed that the degradation rate of biodegradable lignin cryogels was significantly higher than that of lignin-free controls (Fig. 4c). LC_1_ cryogels exhibited a maximum mass loss of 32.7% after 130 days of soil incubation, in contrast to 9.3% for NC_0_ cryogels. This enhanced degradation, 3.5 greater than that of the control, indicates that lignin incorporation increased the biodegradability of the cryogel. Studies suggest that lignin-containing materials can undergo degradation through the activity of ligninolytic microorganisms and associated oxidative enzymes. Therefore, these processes may have contributed to the enhanced degradation observed here, although further microbial and enzymatic analysis would be required to confirm this mechanism.^24,41^ The degradation process was characterized by a biphasic pattern initially, degradation was gradual (25.3% additional mass loss), consistent with progressive microbial colonization and structural degradation.^42–44^ Importantly, such a regulated degradation profile positions cryogels as effective bioreactors, maintaining structural and functional stability during critical crop developmental stages while concurrently degrading to yield environmentally beneficial components. This degradation aligns with the functional lifespan of crops (3–6 months), facilitating continuous regulation of water and nutrient retention as the crops develop. The multifunctional performance observed in LC_1_ demonstrates that lignin incorporation simultaneously enhances mechanical stability, biodegradability, and hydration behavior without compromising environmental compatibility. These synergistic improvements establish a direct structure–function relationship in which lignin-mediated modifications of cryogel architecture and chemistry translate into enhanced agricultural functionality. Lignin-based cryogels represent a promising class of biodegradable rhizosphere-engineering materials capable of improving soil-water management while supporting sustainable and climate-resilient crop production. Lignin incorporation simultaneously enhances the mechanical strength and elasticity of the cryogels while promoting their biodegradation in soil, making them promising sustainable materials for agricultural and environmental applications.

Biodegradable soil-conditioning materials based on lignin, cellulose, and alginate have shown considerable potential for improving soil-water availability and plant performance (Table S2). For example, a lignin/konjac glucomannan/sodium alginate hydrogel exhibited a water absorption capacity of 41.23 g g^−1^, approximately 20% soil-burial degradation after 120 days, and improved tobacco performance under drought stress.^45^ An alkali-lignin hydrogel exhibited a swelling ratio of 2013% and increased soil water retention across different soil textures^46^ while, cellulose-derived hydrogels have similarly improved soil moisture retention and crop growth.^47^ Macroporous alginate hydrogels have also increased substrate moisture retention and supported lettuce growth under drought stress.^48^ Against this background, the present LC_1_ cryogel combined sustained water-retention behavior with 32.7% mass loss after 130 days of soil burial and improved germination, growth, and physiological performance across maize, wheat, and rapeseed under greenhouse and field conditions. Rather than demonstrating superiority in any single parameter, these findings highlight the integrated water-regulating, biodegradable, mechanically resilient, and agronomically functional characteristics of the lignin-derived cryogel.

The analysis of water absorption and retention revealed that lignin concentration significantly influences the hydration behavior and thermal stability of cryogels (Fig. 5). At 35 °C, LC_1_ demonstrated the highest water absorption capacity, markedly surpassing both LC_02_ and NC_0_. This enhanced water uptake can be attributed to the high density of hydroxyl and phenolic functional groups present in lignin, which facilitate hydrogen bonding with water molecules, thereby increasing the hydrophilicity of the matrix. Furthermore, the interconnected porous architecture of LC_1_ promotes rapid water diffusion and storage within the cryogel network. LC_1_ maintained its superior hydration capacity at elevated temperatures of 45 °C and 65 °C, exhibiting significant thermal resilience (Fig. 5a–d). This thermal stability suggests that lignin contributes to the rigidity of polymer networks, thereby preventing pore collapse when exposed to heat and maintaining water storage capabilities under varying temperatures and environmental conditions. At 75 °C, LC_1_ maintained a mean relative mass retention (*M*_r_) of approximately 55% after 12 days, compared with approximately 42% for LC_02_ and 12% for NC_0_ (Fig. 5d), demonstrating the greater water-retention capacity of the lignin-containing LC1 formulation under elevated-temperature conditions). This thermal resistance indicates that lignin not only enhances water uptake but also preserves the pore structure of cryogels against heat-induced degradation, thereby ensuring moisture availability under challenging environmental conditions. This characteristic is particularly relevant in agricultural soils, which often experience elevated surface temperatures, where conventional hydrogels typically lose their efficacy. These findings position lignin-based cryogels, particularly LC_1_, as mechanically robust, thermally resistant, and biodegradable biomaterials capable of sustaining water supply and facilitating plant growth across diverse environmental conditions. This multifunctional performance underscores a mechanistic relationship between material structure, rhizosphere activity, and physiological enhancement in plants, thereby validating lignin cryogels as a promising advancement in sustainable soil conditioning systems for climate-resilient agriculture.

**Fig. 5:**
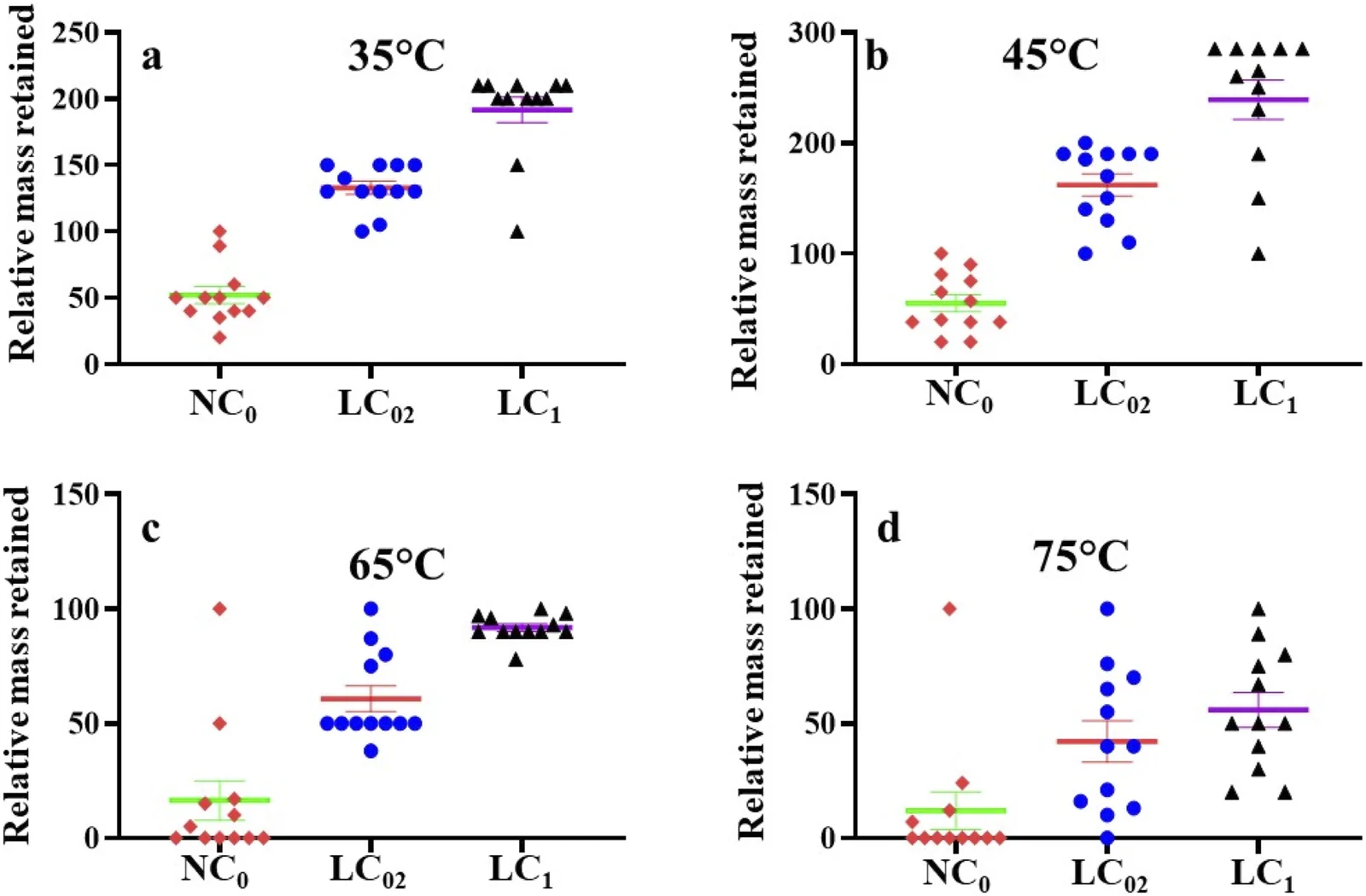
Water-retention behavior of cryogel formulations NC_0_, LC_02_, and LC_1_ during 12 days of incubation at 50% relative humidity under different temperatures. (a) 35 °C, (b) 45 °C, (c) 65 °C, and (d) 75 °C. Water retention is expressed as the relative mass retained (*M*_r_, %), calculated as the percentage of the initial hydrated mass remaining at each measurement time. This parameter represents water-retention behavior during drying and is distinct from the water-uptake percentage (*W*_p_) defined in eqn (2).

### 3.3 Biocompatibility and phytostimulatory effects

The phytotoxicity and biocompatibility of lignin-cryogels were evaluated using the germination index (GI) together with early seedling physiological responses following exposure to NC_0_, LC_02_, and LC_1_ (Fig. S2a and b). The GI integrates seed germination and root elongation and is therefore widely used to assess material-associated phytotoxicity.^49^ According to established classifications, GI values <50% indicate severe phytotoxicity to established classifications, GI values <50% indicate severe phytotoxicity, values of 50–80% indicate moderate phytotoxicity, and values >80% indicate the absence of substantial phytotoxic effects.^50^ Both lignin-containing formulations showed GI values above the control level, with LC02 and LC1 reaching approximately 115% and 125%, respectively, compared with ∼100% for the control (Fig. S2a). In contrast, NC0 exhibited a GI of approximately 90%, whereas the synthetic hydrogel (St) showed a markedly lower GI of approximately 30% (*P* < 0.01). These results indicate that LC_02_ and LC_1_ were non-phytotoxic and supported early seedling establishment. GI values exceeding 100% reflect greater germination and/or root elongation relative to the control and are therefore consistent with a potential phytostimulatory response. However, values above 100% may also be influenced partly by inherent biological and experimental variability among replicates. Accordingly, these values should be interpreted cautiously and in conjunction with the broader physiological responses rather than as definitive evidence of biostimulation alone.

The lower GI observed for St indicates substantially poorer compatibility with early seedling development under the tested conditions. Although residual monomers or degradation products have been proposed as potential contributors to the phytotoxicity of some synthetic polymeric materials^24,51^ their involvement was not directly examined here. Therefore, the present results demonstrate an inhibitory response to St under the experimental conditions but do not establish its underlying chemical mechanism. Similarly, the higher GI values of LC02 and LC1 relative to NC0 suggest that lignin incorporation was associated with improved phytocompatibility, although GI alone cannot establish a direct causal mechanism.

The physiological responses further supported the favorable performance of the lignin-containing cryogels (Fig. S2b). LC_1_ produced the strongest overall responses across root and shoot growth, biomass accumulation, chlorophyll content, and plant water status, followed by LC_02_, whereas NC_0_ showed intermediate responses and St generally exhibited the lowest values. The progressive improvement from NC_0_ to LC_02_ and LC_1_ was consistent with the increasing lignin content of the formulations. These responses, together with the structural and water-retention characteristics of the cryogels, suggest that lignin incorporation contributed to a material environment favorable for early seedling growth. Enhanced water availability and the physicochemical properties of the lignin-containing matrix may contribute to these responses, although the specific biological mechanisms require further investigation.

The GI and physiological data demonstrate that the lignin-containing cryogels, particularly LC1, were highly compatible with early seedling development and showed potential phytostimulatory effects under the tested conditions. Importantly, the GI values >100% for LC_02_ and LC_1_ should be considered supportive, rather than conclusive, evidence of biostimulation because experimental and biological variability may also contribute to values above the control baseline. These findings are consistent with previous reports describing the biodegradability and biological compatibility of lignin-based biomaterials^1^ and support the potential of lignin-containing cryogels as sustainable soil-conditioning materials for agricultural applications.

### 3.4 Rhizosphere engineering promotes seedling establishment

The enhancement of seed germination and early plant development observed in lignin-based cryogel treatments is fundamentally governed by material architecture and functional chemistry, which collectively regulate rhizosphere hydration and nutrient accessibility, facilitating capillary-driven water transport and efficient root penetration. From a materials science perspective, such hierarchical porosity increases surface area and water-holding capacity, enabling dynamic moisture buffering within the soil matrix. FTIR characterization confirmed the presence of hydrophilic functional groups, including hydroxyl and carboxyl moieties, which promote hydrogen bonding and water absorption. At the physiological level, sustained hydration directly influences seed metabolic activation, enzymatic mobilization of storage reserves, and radicle elongation, thereby accelerating germination kinetics and seedling establishment. These physicochemical properties establish lignin cryogels as functional rhizosphere modulators rather than passive soil additives.^52,53^ Thermal analysis further revealed that the incorporation of lignin regulates hydration–dehydration dynamics. Such controlled water delivery stabilizes seed osmotic balance and reduces dehydration-induced oxidative stress during early growth, which is a key physiological constraint under nutrient-limited or drought-prone conditions. Greenhouse experiments conducted across MZ, WT, and RP species confirmed the biostimulatory capacity of lignin cryogels under nutrient-limited conditions (Fig. 6a–f). The LC_1_^-nv^ treatment (0.1 wt%) produced the most pronounced response, resulting in a 65.4% increase in shoot length in MZ relative to the control (NC_0_^-nv^; *F* (5, 84) = 19.62, *P < 0.0001). WT and RP also exhibited significant growth stimulation, with shoot elongation increases of 42.8% and 31.6%, respectively. Even the lower-dose treatment (LC_02_^-nv^, 0.02% w/w) produced statistically significant growth enhancement (*P* < 0.05), indicating a clear dose–responsive relationship.

**Fig. 6:**
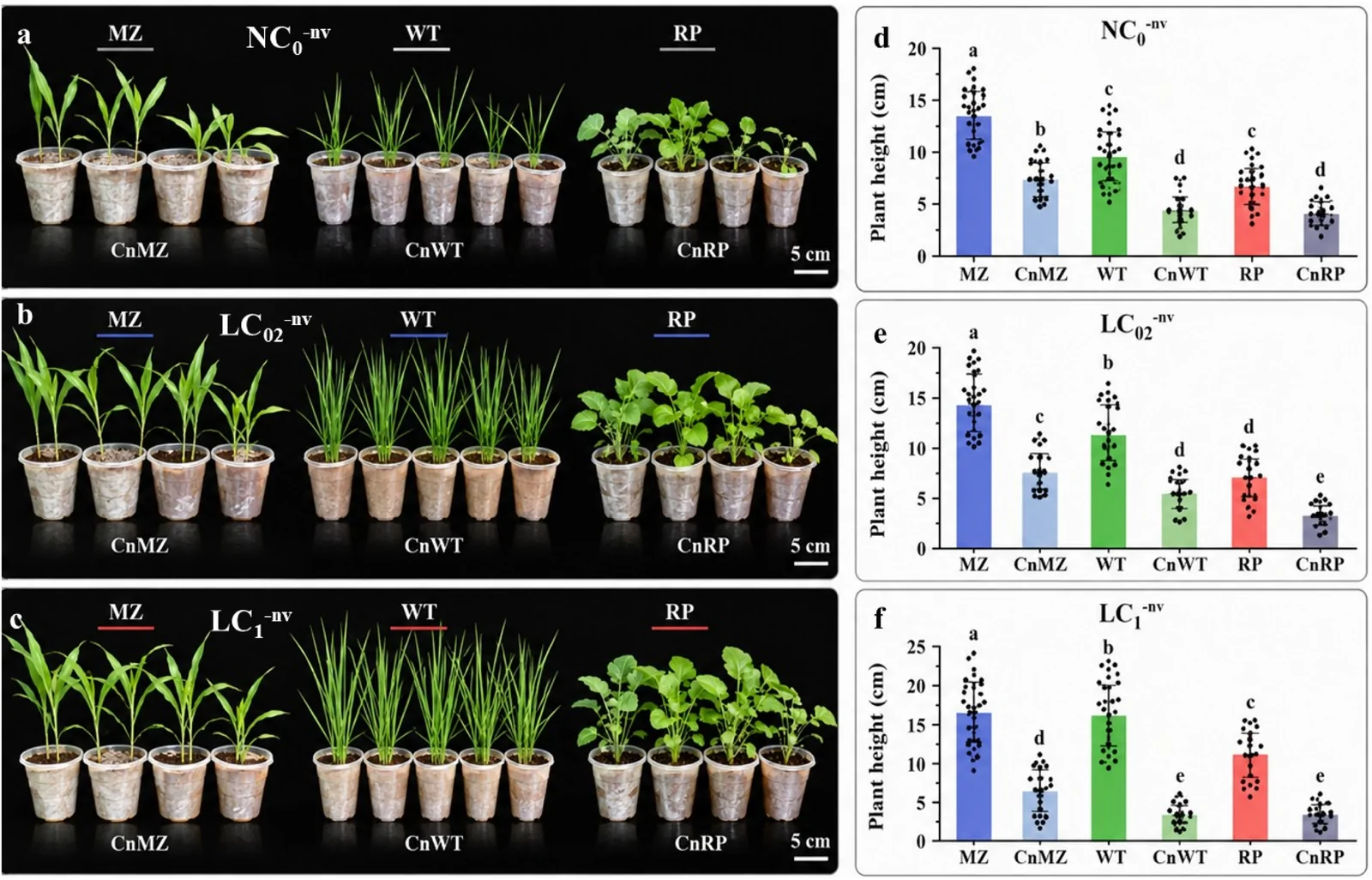
Effect of lignin-based cryogel formulations on early plant growth under controlled (nv) conditions across multiple crop species. (a–c), representative images showing plant growth following treatment with (a) NC_0_^-fd^, (b) LC_02_^-fd^, and (c) LC_1_^-fd^. Control = untreated plants without cryogel amendment; CnMZ, CnWT, and CnRP = comparative control groups corresponding to maize, wheat, and rapeseed, respectively; MZ = maize; WT = wheat; RP = rapeseed. (d–f) Quantitative analysis of plant height in MZ, WT, and RP under controlled conditions following application of (d) NC_0_^-nv^, (e) LC_02_^-nv^, and (f) LC_1_^-nv^ cryogels. Bars indicate the mean plant height ± standard deviation, with individual data points representing biological replicates (*n* ≥ 15).

Morphological observations supported quantitative measurements, as LC_1_^-nv^ treatments increased leaf expansion, shoot biomass accumulation, and overall plant vigor (Fig. 6d–f). These phenotypic responses suggest enhanced photosynthetic capacity and improved early carbon assimilation efficiency, likely resulting from sustained moisture availability and improved nutrient diffusion within the cryogel-modified soil matrix. In contrast, treatments lacking lignin exhibited reduced germination vigor and limited seedling development, thereby confirming the functional contribution of lignin incorporation.

Lignin cryogel significantly enhanced germination performance under field conditions. Germination percentages and seedling establishment rates exhibited substantial increases within the first seven days for the LC_02_^-nv^ and LC_1_^-nv^ treatments when compared to the NC_0_^-nv^ controls. After 20 days, seedlings treated with cryogel demonstrated improved root system development and enhanced soil moisture retention capacity. These findings suggest that cryogels serve not only as water reservoirs but also as physical scaffolds that facilitate root anchorage and promote early rhizosphere stabilization. Quantitative assessments of growth revealed consistent increases in plant height and leaf length across all species following the application of cryogels. By day 21, maximum plant heights reached 23 cm in MZ, 18 cm in WT, and 7 cm in RP under lignin cryogel treatment, consistently surpassing the performance of lignin-free controls. These enhancements were associated with increased node formation and canopy expansion, indicating a heightened rate of vegetative development.

### 3.5 Field-scale validation of cryogel-enabled crop growth enhancement

The translational performance of lignin cryogels was validated through field trials, where the LC_1_^-fd^ treatment consistently demonstrated superior efficacy compared to LC_02_^-fd^ and NC_0_^-fd^ across all tested crops (Fig. S3a–f). MZ exhibited the most pronounced response, with the LC_1_^-fd^ treatment resulting in a 59.2% increase in plant height relative to the NC_0_^-fd^ controls (*F* (5,108) = 23.78, *P* < 0.0001). WT and RP displayed similar growth patterns, with post hoc analyses confirming statistically significant inter-treatment differences (*P* < 0.001).

The application of cryogels significantly enhanced canopy architecture, leading to increased leaf density and vertical biomass accumulation (Fig. S3a–f). This canopy expansion is likely attributable to two primary soil-modifying mechanisms: (i) cryogel-mediated moisture stabilization, which mitigates drought-induced growth fluctuations, and (ii) enhanced soil aeration facilitated by the porous cryogel network, which promotes oxygen diffusion and improves root respiration efficiency. Notably, no phytotoxic effects were observed at any tested concentration (0–0.1 wt%), thereby affirming the biological compatibility of lignin cryogels. The strong correlation between greenhouse and field results (*R*^2^ = 0.89 for MZ plant height) underscores the environmental robustness and scalability of cryogel-mediated growth enhancement. Seed germination assays further confirmed that lignin cryogels function as growth-supporting soil amendments by enhancing moisture retention and creating a protective microenvironment around seeds. The hydrophilic and biodegradable characteristics of the lignin–starch composite matrix facilitate gradual hydration and support microbial colonization, both of which are recognized as critical factors in early plant establishment.^52–55^ Additionally, starch components confer biodegradable swelling capacity, thereby enhancing soil structure and water retention properties.^18,56^ Overall, lignin-based cryogels exhibited a pronounced dose-dependent effect on germination, seedling growth, and canopy development across various crop species. The incorporation of structural porosity, hydrophilic functional groups, and biodegradable polymer networks permits these cryogels to serve as multifunctional soil conditioners, facilitating early plant establishment and promoting sustainable crop production in nutrient-limited environments.

### 3.6 Seedling growth and biomass enhancement mediated by lignin-based cryogels

The availability of soil water and rhizosphere hydration significantly influences early seedling establishment. The cryogels utilized in this study, derived from lignin, possess interlocked macropore structures and exhibit high hydrophilicity due to the presence of hydroxyl and carboxyl groups on their surfaces. These features facilitate rapid water absorption and sustained moisture release. The structural design enables the cryogel to function as an active reservoir for rhizosphere hydration, thereby stabilizing the soil-plant water continuum and supporting prolonged physiological activity during the critical phase of seedling development. Consistent with this mechanism, lignin cryogels markedly enhanced leaf elongation in maize (MZ, WT, and RP) when compared to the NC_0_ control (Fig. 7a–f). The LC_1_ formulation demonstrated the most pronounced growth response under controlled conditions, with maize seedlings achieving an average height of approximately 23 cm over 21 days, in contrast to 13 cm in the NC_0_ group. Similar responses observed in wheat and rapeseed indicate that the growth-promoting effects of lignin cryogels are conserved across monocot and dicot species. Importantly, this enhancement suggests a causal relationship between the structural characteristics of the cryogel material and the physiological performance of plants, rather than a mere amendment effect on soil. The macroporous structure improves water retention within the soil and enhances root-soil interfacial contact, thereby creating a continuous water supply and facilitating nutrient diffusion to actively growing roots. This prolonged hydration likely sustains cellular turgor pressure, promotes irreversible cell swelling, leaf expansion, and biomass accumulation, which are critical indicators of early plant vigor. Prior research has demonstrated that porous hydrogel matrices enhance the availability of rhizosphere water and improve nutrient acquisition efficiency, thereby augmenting plant growth performance.^57^ Components derived from lignin have been reported to regulate plant developmental signaling and stress–response pathways.^58^ Although molecular signaling was not directly quantified in this study, the observed uniform dose-dependent response strongly suggests that hydration-mediated physiological processes are the primary drivers of enhanced growth. The LC_1_^-fd^ treatment underscores the functional stability of lignin cryogels under environmentally relevant conditions, which accounts for its consistent performance in generating the highest leaf elongation across all species, with maize and rapeseed exhibiting mean leaf lengths of approximately 20–21 cm (Fig. 6f and 7d).

**Fig. 7:**
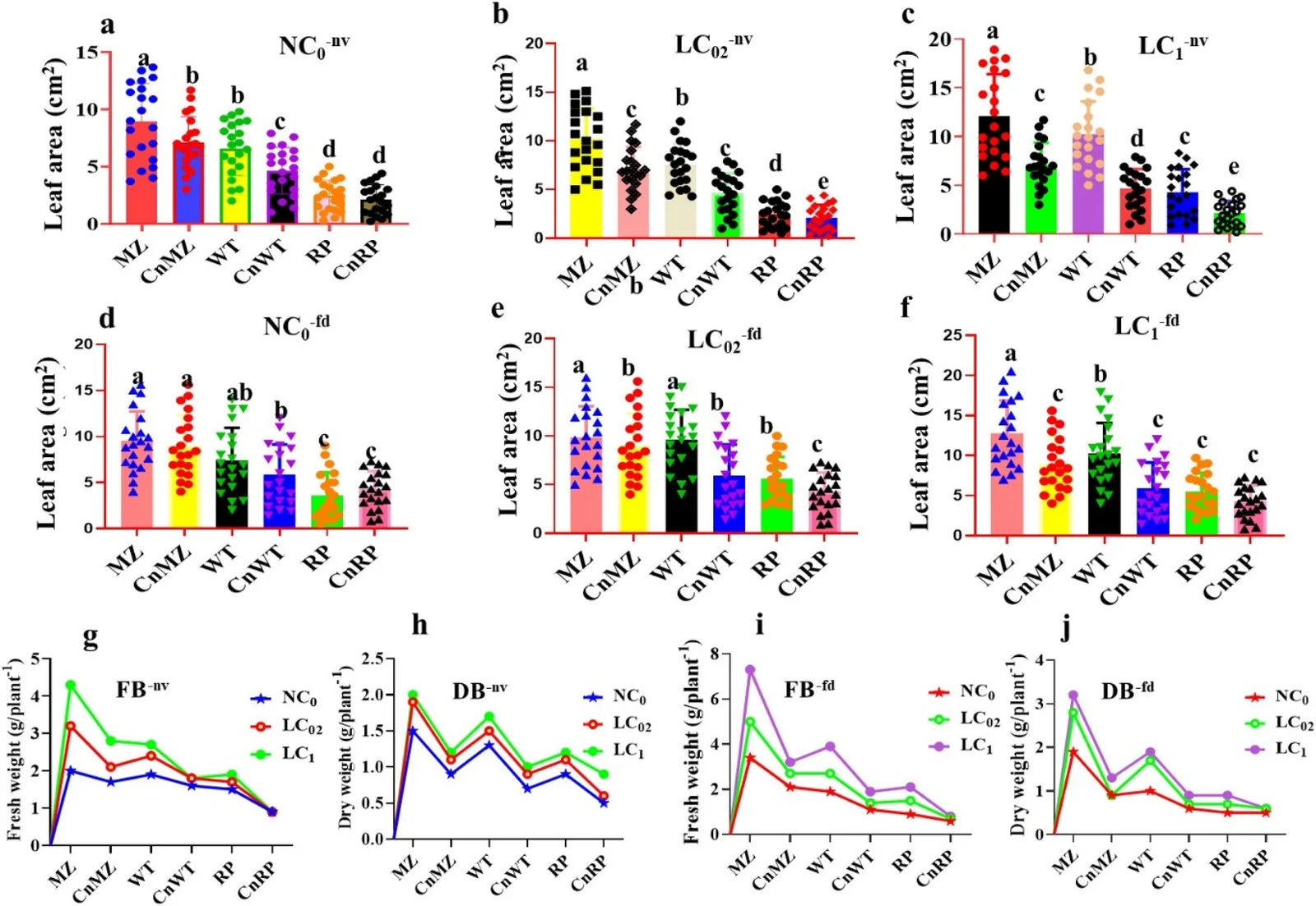
Effect of lignin-based cryogel formulations on leaf elongation and biomass accumulation across multiple crop species under nv and fd conditions. (a–c) Leaf area responses under controlled conditions following treatment with NC_0_^-nv^, LC_02_^-nv^, and LC_1_^-nv^ cryogels. (d–f) Corresponding field performance under NC_0_^-fd^, LC_02_^-fd^, and LC_1_^-fd^ treatments. Data mean leaf length ± standard error with individual biological replicates displayed. Effect of cryogel treatments at varying concentrations on biomass accumulation under nv and fd conditions. Note: (g) fresh biomass in nv conditions (FB^-nv^); (h) dry biomass in nv conditions (DB^-nv^); (i) Fresh biomass in fd conditions (FB^-fd^); and (j) dry biomass in fd conditions (DB^-fd^). Lignin-containing cryogels exhibited concentration-dependent growth enhancement across all species, with LC_1_ consistently producing the strongest leaf elongation response, indicating improved rhizosphere hydration and early plant development.

The consistent performance of lignin cryogels under varying environmental conditions demonstrates their role as environmental buffering mechanisms that mitigate temporary water scarcity and support continued physiological growth. This stabilization of moisture within the rhizosphere is particularly critical in fields experiencing moisture fluctuations, which often hinder the establishment and development of crops in their early stages and subsequently limit yield potential. Statistical analyses have confirmed the presence of significant treatment effects across different species and environments, revealing a dose-dependent functional variance among the various formulations of cryogel. Importantly, no phytotoxic effects were observed, indicating the biological compatibility of these materials. These structural and physiological advantages translated directly into enhanced biomass accumulation (Fig. 7g–j). Notably, the LC_02_^-nv^ and LC_1_^-nv^ treatments produced significantly greater fresh biomass compared to LC_02_^-nv^, reaching up to 3.80 g of fresh biomass across a range of genotypes (Fig. 7g), while LC_02_^-nv^ exhibited the highest dry biomass at approximately 2.2 g (Fig. 7h). This amplification of hydration provided by cryogels suggests that their application not only facilitates short-term water uptake but also enhances long-term carbon fixation and biomass development. Conversely, the NC_0_ treatment consistently resulted in lower biomass levels, underscoring the necessity of lignin incorporation to ensure the stability of the cryogel.

The LC_1_^-fd^ treatment significantly enhanced the fresh and dry biomass of all species under field conditions (Fig. 7i and j). Such notable performance suggests that an increase in lignin levels will enhance structural stability and extend the functional lifespan, thereby ensuring that the rhizosphere remains hydrated during critical growth periods. The ability to maintain a consistent water supply over extended durations offers a functional advantage compared to traditional soil amendments, which often fail under prolonged environmental stress. These findings align with previous studies indicating that hydrogel-based materials improve plant productivity by enhancing soil water retention and nutrient availability.^53,59^ Facilitating hydration, the incorporation of lignin is likely to enhance nutrient dynamics within the rhizosphere; lignin-derived materials have been shown to improve the availability of essential micronutrients, such as Zn^2+^, Mg^2+^, and Cu^2+^, thereby promoting photosynthetic processes and metabolic activities.^54^ The transformation of lignin into humic-like compounds enhances soil aggregation, cation-exchange capacity, and nutrient retention.^60,61^

These physicochemical enhancements create a more conducive rhizosphere environment that improves nutrient uptake efficiency and supports sustained plant growth. The improved root growth facilitated by hydration mediated through the hydrogel has also been associated with increased stress tolerance and enhanced plant productivity. The current results establish a direct mechanistic relationship between the structure of lignin cryogel materials and their functional impact on the rhizosphere, physiological growth of plants, and biomass production. The cryogel functions as an active interface material for soil and plants, regulating water supply, stabilizing physiological activities, and increasing plant yield. This synergistic relationship between material and plant functionality represents a significant advancement over traditional hydrogels, which primarily serve as passive water reservoirs without enhancing physiological functions. Sustainably, biodegradable soil-conditioning materials can be developed from lignocellulosic waste, thereby reducing environmental persistence and promoting bioeconomic circulation strategies. These findings position lignin-based cryogels as innovative multifunctional biomaterials capable of addressing water scarcity issues and mitigating its detrimental effects on crop growth, thereby contributing to a more sustainable agricultural system.

### 3.7 Lignin cryogel amendment modulates root growth and respiration

Root system architecture is a primary determinant of early plant establishment, as it governs water acquisition, nutrient uptake, and subsequent shoot productivity. To ascertain whether the incorporation of lignin cryogel alters root developmental dynamics, we quantified root length, dry weight, and fresh weight across WT, MZ, and RP under both conditions using three cryogel formulations (NC_0_, LC_02_, and LC_1_) (Fig. S1). Under greenhouse conditions, both LC_02_^-nv^ and LC_1_^-nv^ treatments modified root growth responses compared to NC_0_^-nv^, although the magnitude and direction of the response varied among species (Fig. S4a–c). LC_02_^-nv^ generally maintained or slightly improved root elongation relative to NC_0_^-nv^, whereas LC_1_^-nv^ produced more variable responses, with some genotypes exhibiting comparable or reduced root length. For instance, maize and wheat exhibited relatively similar root elongation under LC_1_^-nv^ compared to NC_0_^-nv^, while rapeseed demonstrated a modest reduction. This pattern suggests that under stable environmental conditions, increasing lignin density does not uniformly stimulate primary root elongation and may instead reallocate resources toward shoot growth and biomass accumulation. Importantly, these findings indicate that enhanced shoot productivity under lignin cryogel treatment does not necessarily require proportional increases in primary root length. Conversely, under field conditions, lignin cryogel treatments, particularly LC_1_^-fd^, consistently promoted root elongation across all tested crops (Fig. S4d–f). Maize roots reached approximately 15 cm under LC_1_^-fd^ compared to ∼11 cm in NC_0_^-fd^, while wheat exhibited a similar increase from ∼8 cm to ∼12 cm. Rapeseed also demonstrated improved root elongation relative to the control. These results illustrate that higher lignin incorporation enhances root extension under environmentally variable conditions, where soil moisture availability and nutrient diffusion are less stable. The stronger field response compared to greenhouse conditions indicates that lignin cryogels provide functional advantages specifically under environmental stress or fluctuation. The differential response between greenhouse and field environments highlights an important functional distinction: lignin cryogels do not act solely as direct stimulants of root growth but instead enhance root system performance in a context-dependent manner linked to environmental constraints. Under controlled irrigation, root elongation remains adequate even without lignin, whereas under field variability, lignin cryogels improve rhizosphere conditions that support continued root extension. This interpretation aligns with the previously observed enhancements in leaf area expansion and biomass accumulation, confirming the coordinated enhancement of both belowground and aboveground growth systems.

Importantly, the lack of consistent root inhibition across species indicates that lignin cryogels do not exert phytotoxic effects at the tested concentrations, thereby supporting their biological compatibility. Instead, the data demonstrate that the incorporation of lignin modifies root development in a manner that enhances overall plant growth outcomes. The observed enhancement of roots under lignin cryogel treatment aligns with previous reports showing that lignin-derived materials and humic-like substances can improve the physicochemical properties of the rhizosphere and nutrient availability.^20,54,62^ However, in contrast to earlier studies that predominantly attribute root stimulation to direct biochemical signaling, the current findings suggest that the primary functional effect is an improvement in environmental buffering, particularly under field conditions. This conclusion is substantiated by the more pronounced response observed in fd treatments compared to nv treatments. These findings establish that lignin cryogels serve as adaptive modifiers of the rhizosphere that enhance root system performance under environmentally relevant conditions, rather than functioning merely as growth stimulants. The coordinated improvements in root elongation, shoot expansion, and biomass accumulation illustrate that lignin cryogels enhance the developmental efficiency of the entire plant. This environment-dependent functionality represents a critical mechanistic characteristic that differentiates lignin cryogels from conventional hydrogel systems and underscores their potential application as sustainable soil-conditioning biomaterials for crop establishment in water-limited or variable agricultural systems. Future research should prioritize understanding the physiological and molecular mechanisms through which lignin-based cryogels influence plant development, particularly regarding their long-term efficacy in enhancing biomass yield and crop quality for sustainable agriculture.

### 3.8 Comparative analysis of physiological responses

To determine whether lignin cryogel influence plant metabolic status beyond morphological growth enhancement, key physiological parameters, including, chlorophyll content, soluble sugar accumulation, and total protein content were evaluated under nv and fd conditions (Table 1). These traits provide insight into photosynthetic efficiency, carbon allocation, and nitrogen assimilation, respectively, and collectively reflect plant functional performance.

Comparative analysis of physiological response; to determine whether lignin cryogel formulations modulate plant physiological performance, total chlorophyll, soluble sugar, and protein contents were quantified under greenhouse (nv) and field (fd) conditions. Across species, LC_1_ consistently produced the strongest physiological enhancement relative to NC_0_, LC_02_, and untreated controls, indicating a dose-dependent response^a^

**Table:** 

Crop	Total chlorophyll (mg g^−1^) – greenhouse (nv)					Total chlorophyll (mg g^−1^) – field conditions (fd)				
	Control	NC_0_^-nv^	LC_02_^-nv^	LC_1_^-nv^	% ↑ and ↓	Control	NC_0_^-fd^	LC_02_^-fd^	LC_1_^-fd^	% ↑ and ↓
MZ	1.55 ± 0.15^a^	1.82 ± 0.16^ab^	2.41 ± 0.20^c^	2.85 ± 0.25^cd^	34.58%	1.42 ± 0.18^a^	1.65 ± 0.21^ab^	2.26 ± 0.22^c^	2.68 ± 0.24^d^	39.43%
CnMZ	1.55 ± 0.15^b^	1.82 ± 0.16^a^	1.95 ± 0.17^a^	2.11 ± 0.18^c^	25.55%	1.42 ± 0.18^b^	1.65 ± 0.21^a^	1.83 ± 0.17^ab^	1.96 ± 0.19^c^	27.46%
WT	1.23 ± 0.12^a^	1.67 ± 0.87^b^	1.85 ± 0.15^d^	2.15 ± 0.18^cd^	44.12%	1.08 ± 0.14^a^	1.48 ± 0.13^b^	1.72 ± 0.18^c^	1.98 ± 0.21^cd^	45.60%
CnWT	1.23 ± 0.12^a^	1.45 ± 0.39^a^	1.57 ± 0.88^bc^	1.64 ± 0.16^a^	32.54%	1.08 ± 0.14^a^	1.24 ± 0.33^a^	1.39 ± 0.58^bc^	1.46 ± 0.19^ab^	35.18%
RP	0.92 ± 0.12^a^	1.32 ± 0.11b	1.45 ± 0.15^d^	1.72 ± 0.18^c^	54.44%	0.85 ± 0.11^a^	1.18 ± 0.14^b^	1.34 ± 0.16^d^	1.61 ± 0.18^c^	47.84%
CnRP	0.92 ± 0.12^b^	1.04 ± 0.11^a^	1.15 ± 0.12^ac^	1.25 ± 0.13^ac^	26.77%	0.85 ± 0.11^b^	0.98 ± 0.13^a^	1.09 ± 0.14^ac^	1.18 ± 0.15^ac^	27.47%

a Control = untreated plants without cryogel amendment; CnMZ, CnWT, and CnRP = comparative control groups corresponding to maize, wheat, and rapeseed, respectively; MZ = maize; WT = wheat; RP = rapeseed. nv: *in vitro* (greenhouse-grown), fd: field conditions, Total Chlr: total chlorophyll, and %↑ and ↓: percentage increased/decreased: percent change relative to control under respective conditions. Superscript letters denote statistically significant differences (*P* < 0.05) based on *post-hoc* Tukey's test.

**Table:** 

Crop	Total soluble sugar – greenhouse (nv)					Total soluble sugar – field conditions (fd)				
	Control	NC_0_^-nv^	LC_02_^-nv^	LC_1_^-nv^	% ↑ and ↓	Control	NC_0_^-fd^	LC_02_^-fd^	LC_1_^-fd^	% ↑ and ↓
MZ	3.52 ± 0.25^c^	4.97 ± 0.23^a^	5.57 ± 0.32^ac^	8.45 ± 0.34^b^	42.78%	3.33 ± 0.30^c^	4.78 ± 0.27^a^	5.36 ± 0.33 ^ab^	8.03 ± 0.40^b^	41.93%
CnMZ	3.52 ± 0.25^c^	4.06 ± 0.23^b^	4.62 ± 0.53^ac^	7.63 ± 0.35^ad^	32.50%	3.33 ± 0.30^c^	3.85 ± 0.29^b^	4.43 ± 0.45^bc^	7.01 ± 0.42^a^	30.24%
WT	3.56 ± 0.21^c^	4.21 ± 0.23^ac^	6.95 ± 0.33^d^	5.83 ± 0.40^bd^	25.45%	3.41 ± 0.26^b^	3.96 ± 0.28 ^ab^	6.48 ± 0.34^d^	5.45 ± 0.43 ^cd^	25.84%
CnWT	3.56 ± 0.21^b^	3.89 ± 0.23^b^	6.21 ± 0.28^c^	9.88 ± 0.61^a^	22.78%	3.41 ± 0.26^b^	3.72 ± 0.27^b^	5.82 ± 0.31^c^	9.16 ± 0.64^a^	22.29%
RP	2.76 ± 2.7^c^	3.21 ± 2.9^a^	5.65 ± 0.30^b^	5.4 ± 0.42^b^	43.56%	2.58 ± 2.6^c^	2.97±2.7^a^	5.32 ± 0.30^b^	5.09 ± 0.38^b^	44.84%
CnRP	2.76 ± 2.7^b^	3.21 ± 2.9^a^	6.18 ± 4.0^c^	7.45 ± 4.8^d^	23.98%	2.58 ± 2.6^b^	2.97 ± 2.7^a^	5.82 ± 3.5^c^	6.91 ± 4.2^d^	24.89%

**Table:** 

Crop	Total protein – greenhouse (nv)					Total protein – field conditions (fd)				
	Control	NC_0_^-nv^	LC_02_^-nv^	LC_1_^-nv^	% ↑ and ↓	Control	NC_0_^-fd^	LC_02_^-fd^	LC_1_^-fd^	% ↑ and ↓
MZ	12.1 ± 0.8^b^	14.3 ± 1.0^d^	15.6 ± 1.3^ac^	18.5 ± 1.5^c^	58.89%	11.4 ± 0.9^b^	13.5 ± 1.2^d^	14.9 ± 1.4^ac^	16.3 ± 1.5^c^	54.3%
CnMZ	12.1 ± 0.8^a^	14.3 ± 1.0^b^	17.8 ± 1.4^d^	19.9 ± 1.5^dc^	64.46%	11.4 ± 0.9^a^	13.5 ± 1.2^b^	16.9 ± 1.3^d^	18.1 ± 1.6^dc^	58.77%
WT	8.2 ± 0.50^a^	9.5 ± 0.6^c^	11.8 ± 0.9^cb^	13.2 ± 1.0^d^	60.98%	7.5 ± 0.45^a^	8.8 ± 0.6^c^	10.9 ± 0.9 ^cb^	12.3 ± 1.1^d^	64.00%
CnWT	8.2 ± 0.50^a^	9.5 ± 0.6^c^	10.1 ± 0.7^c^	11.3 ± 0.9b^c^	37.80%	7.5 ± 0.45^a^	8.8 ± 0.6^c^	9.3 ± 0.6^c^	10.4 ± 0.9^bc^	38.67%
RP	11.3 ± 1.0^a^	13.0 ± 1.2^c^	17.5 ± 1.5^ab^	19.4 ± 1.5^d^	71.68%	10.5 ± 1.1^a^	12.1 ± 1.3^c^	16.5 ± 1.2^ab^	18.0 ± 1.5^d^	71.43%
CnRP	11.3 ± 1.0^a^	13.0 ± 1.2^c^	16.8 ± 1.25^d^	18.3 ± 1.18^b^	61.97%	10.5 ± 1.1^a^	12.1 ± 1.3^c^	15.8 ± 1.0^d^	17.2 ± 1.3^b^	63.81%

### 3.9 Chlorophyll content

Lignin-containing cryogel, particularly LC_1_, consistently increased chlorophyll content across all crop species relative to NC_0_ and LC_02_ treatments under both nv and fd conditions. Under controlled conditions, LC_1_ treatment resulted in chlorophyll increases of 34–44% in MZ and WT, while similar enhancement was maintained under field conditions (39.43–45.60%). RP also exhibited a pronounced response, with increases approaching 47.84% (Table 1). These increases in chlorophyll content indicate improved photosynthetic capacity, likely arising from enhanced rhizosphere hydration and nutrient availability mediated by the cryogel matrix. Adequate soil moisture stabilizes chloroplast integrity and supports nitrogen assimilation, which is essential for chlorophyll biosynthesis.^63^ The consistency of chlorophyll enhancement across both greenhouse and field environments suggests that lignin incorporation improves the functional stability of the cryogel under environmental variability. Notably, WT showed the highest chlorophyll increase under both nv (44.12%) and fd (45.60%) conditions, indicating species-specific responsiveness. However, all tested crops demonstrated positive responses relative to lignin-free controls, confirming the broad applicability of the material.

### 3.10 Soluble sugar accumulation

Soluble sugar content increased significantly in response to LC_1_ treatment across species and environments. Under nv conditions, MZ and RP exhibited the strongest increases (approximately 42.78–43.56%) (Table 1), with similar magnitudes maintained under field conditions. WT and CnMZ also showed moderate but consistent increases relative to controls. Soluble sugar function both as metabolic substrates and osmoprotectants, contributing to stress buffering and growth regulation.^64^ The elevated sugar level observed in cryogel treated plants suggest improved carbon assimilation efficiency and osmotic adjustment capacity, likely facilitated by stabilized soil moisture and enhanced nutrient diffusion. These findings align with previous reports indicating that moisture-retaining biopolymeric soil conditioners enhance carbohydrate allocation and stress tolerance in plants.^65^ Impotently, the reproducibility of sugar accumulation trends under both nv and fd conditions demonstrates that the physiological benefits of LC_1_ are not restricted to controlled environments but extend to natural field variability.

### 3.11 Protein content

The total protein increased significantly in plants treated with LC_1_, with increases ranging from 37.80% to 71.68% under non-volatile conditions (Table 1), and the greatest response was observed in RP. Protein accumulation is closely associated with nutrient availability and metabolic activity during plant growth. The progressive increase across the cryogel treatments therefore suggests that improved rhizosphere conditions, particularly moisture retention and nutrient availability, supported metabolic processes associated with protein accumulation. More broadly, optimization of the growth environment has been shown to enhance cellular protein accumulation; for example, optimized light conditions increased protein content in *Chlorella pyrenoidosa*, illustrating the responsiveness of protein metabolism to favorable environmental regulation.^66^ In the present system, the cryogels are unlikely to directly regulate protein biosynthetic pathways; rather, their effects may arise indirectly through improved soil moisture and nutrient availability around the roots. RP exhibited the strongest protein response under both conditions, indicating a comparatively greater physiological response to cryogel-mediated modification of the rhizosphere.

### 3.12 Integrated physiological interpretation

In all measured parameters, the LC_1_ treatment exhibited the most consistent and significant physiological improvements compared to NC_0_ and LC_02_. The enrichment of chlorophyll indicates an enhancement in photosynthetic potential; elevated soluble sugar levels suggest improvements in carbon metabolism and osmotic balance; increased protein levels reflect better nitrogen assimilation. Collectively, these physiological responses support the hypothesis that lignin-based cryogel functions as a rhizosphere-regulating biomaterial, which stabilizes water availability, enhances nutrient suffusion, and sustains metabolic activity during the initial stages of plant growth. While RP showed the strongest responses in terms of sugar and protein levels, and WT exhibited the greatest improvement in chlorophyll content, all species demonstrated a positive response to the LC_1_ treatment. This suggests that lignin cryogels do not exert species-specific effects but rather provide broad-spectrum physiological benefits. From a sustainability perspective, the ability of lignin-based biomaterials to enhance physiological resilience under changing environmental conditions positions them as effective biodegradable soil-conditioning agents in low-input and climate-adaptive agricultural systems. Further investigations into the physiological modulation and associated molecular pathways resulting from cryogel-mediated physiological changes, incorporating transcriptome profiling and analyses of the rhizosphere microbiome, will enhance our understanding of the underlying molecular mechanisms driving physiological modulation.

### 3.13 Mechanistic validation of cryogel-mediated physiological enhancement

To investigated the physiological basis of cryogel-mediated growth enhancement, we assessed soil water-holding capacity (WHC), moisture retention dynamics, leaf SPAD index, nitrogen content, and key biochemical traits. Application of the optimized LC_1_ formulation significantly increased soil WHC by 42–50% relative to untreated soil, both before and after the greenhouse and field experiments. The enhanced hydration capacity of LC_1_ was likely associated with the combined effects of lignin-derived hydroxyl groups and the interconnected macroporous network formed during cryogelation. These features provide abundant water-binding sites and promote capillary water storage, thereby improving moisture retention within the rhizosphere. LC_1_ treatment also significantly increased leaf SPAD values, which were strongly correlated with extracted chlorophyll concentrations (Table 1), confirming that SPAD measurements reliably reflected treatment-associated changes in leaf chlorophyll status. Leaf nitrogen content was likewise markedly higher in LC_1_-treated plants and positively correlated with protein accumulation (Table 1), linking improved plant nitrogen status with enhanced protein synthesis. Moreover, soil WHC was positively correlated with both chlorophyll and soluble sugar concentrations (Table 1), supporting an association between improved rhizosphere water availability and plant carbon metabolism. Collectively, these relationships indicate that the water-retention capacity of LC_1_ was closely coupled with improved plant physiological and biochemical performance. Two-way ANOVA further identified significant treatment × environment interactions, indicating that the magnitude of the treatment response varied between greenhouse and field conditions. Nevertheless, LC1 consistently produced positive physiological responses across both environments, demonstrating that its beneficial effects were maintained despite environment-dependent variation in response magnitude. Although, lignin-derived cryogels improved soil water retention and crop performance, their effects on nutrient acquisition, rhizosphere microbial dynamics, and root molecular responses remain unresolved. Future studies should integrate microbiome, nutrient-flux, and transcriptomic analyses and examine how cryogels interact with soil temperature and microbial communities to influence carbon cycling under changing climatic conditions.^67^ Long-term, multi-environment field trials are also required to establish their agronomic durability and applicability in climate-resilient agriculture.

## 4 Conclusions

This study demonstrates the potential of lignin-derived cryogels as multifunctional, biodegradable materials for rhizosphere engineering. Lignin incorporation generated interconnected macroporous structures with improved water retention, mechanical stability, and biodegradability, accompanied by enhanced germination, biomass accumulation, and physiological performance across multiple crops under greenhouse and field conditions. However, the mechanisms underlying these plant responses, particularly nutrient acquisition, rhizosphere microbial dynamics, and molecular regulation, were not directly investigated. Future studies integrating microbiome, nutrient-flux, and molecular analyses with long-term, multi-environment field trials are therefore required to establish the mechanisms, durability, and broader agronomic applicability of these materials. Overall, this study provides a structure–function framework for developing lignin-based soil-conditioning materials that link biomass valorization with sustainable and climate-resilient agriculture.

## Author contributions

Nisar Uddin: formal analysis, investigation, writing – original draft, writing – review & editing. Yanwei Xie and Jinxi Zhang: formal analysis, investigation, and review of the original draft. Muhammad Wajid Ullah, Xin Xie and Daochen Zhu: Review & editing, resources: supervision.

## Conflicts of interest

The authors declare that they have no known competing financial interests or personal relationships that could have appeared to influence the work reported in this paper.

## Supplementary Material

RA-OLF-D6RA05329G-s001

## Data Availability

The datasets generated and/or analyzed during the current study are available from the corresponding author upon reasonable request. Should any additional information or clarification be needed, interested parties are encouraged to contact the corresponding author directly. Supplementary information (SI) is available. See DOI: https://doi.org/10.1039/d6ra05329g.
