# Supplementary material for: Lignin-derived biodegradable cryogels engineer the rhizosphere to enhance water availability and crop growth
Source: RSC Adv. 2026 Nov 9. Online ahead of print. doi: 10.1039/d6ra05329g (PMC13746917; doi:10.1039/d6ra05329g)
Supplement: Supplementary file 1 [file RA-OLF-D6RA05329G-s001.pdf]

Supplementary Information (SI) for RSC Advances.
This journal is © The Royal Society of Chemistry

Supplementary materials

Lignin-derived biodegradable cryogels engineer the rhizosphere to enhance water availability and crop growth

Nisar Uddin[1,2], Muhammad Wajid Ullah[3], Yanwei Xie[1], Jinxi Zhang[1], Xie Xin[1*], Daochen Zhu[2*]

1-College of Agriculture, Guizhou University, Guiyang, 550025, China

2-International Joint Laboratory on Synthetic Biology and Biomass Biorefinery, Biofuels Institute, School of the Environment and Safety Engineering, Jiangsu University, Zhenjiang 212013, China

3-Department of Pulp & Paper Engineering, College of Light Industry and Food Engineering, Nanjing Forestry University, Nanjing 210037, China

***Correspondence**

Prof. Dr. Xie Xie

College of Agriculture, Guizhou University

Guiyang, China

Email: xxie1@gzu.edu.cn

Prof. Dr. Daochen Zhu
Email: dczhucn@ujs.edu.cn

Jiangsu University, Zhenjiang, China

Table S1. Major FTIR absorption bands and their assignments in the lignin-based cryogel system

Wavenumber/region (cm^{-1})	Principal assignment	Major structural contribution	Interpretation
3276–3280	O–H and N–H stretching	Lignin hydroxyl groups and keratin	Broad band consistent with hydrogen-bonded hydroxyl and amino groups
∼2930	C–H stretching	Aliphatic groups of lignin and cryogel components	Indicates retention of aliphatic structural moieties
∼1600–1700	C=O stretching / amide I	Primarily keratin peptide groups, with carbonyl contributions from the composite matrix	Characteristic of the protein-containing cryogel framework
∼1500–1600	Aromatic skeletal C–C vibrations / amide II contributions	Lignin aromatic structures and keratin	Supports the presence of lignin aromatic functionality within the composite
∼1000–1300	C–O stretching / amide III contributions	Lignin alcohol/ether groups and keratin	Reflects oxygen-containing lignin functionalities and the proteinaceous matrix

Table S2. Comparison of the present lignin-based cryogel with representative bio-based and biodegradable soil-conditioning hydrogels reported in the literature

Material/system	Performance	Biodegradation	Plant/agronomic response	Ref.
Lignin/konjac glucomannan/sodium alginate hydrogel	Maximum water absorption: 41.23 g g^{-1}; increased soil available water capacity	∼20% degradation after 120 d in soil	Improved photosynthetic performance and drought response of tobacco; prolonged growth period	1
Alkali lignin-based hydrogel	Swelling ratio: 2013%; improved	Not the principal	Primarily evaluated for soil-water regulation	2

	water retention in silt loam and loamy fine sand	reported endpoint		
NaCMC/HEC biodegradable cellulose hydrogel	Improved soil porosity and water retention; plant water-use efficiency increased by 25.25–45.52%	Biodegradable material	Improved germination/growth parameters and photosynthetic performance in cucumber, tomato, and mung bean	3
Macroporous alginate-based hydrogel	Increased substrate moisture retention under drought	Bio-based alginate network	Improved lettuce growth and biomass under drought stress	3
Cellulose-based hydrogel	Maximum swelling ~2500%; 1 wt% hydrogel increased soil water retention by ~20%	Biodegradable within 90 days	Designed for agricultural water retention and fertilizer delivery	4
CMC/MFC biodegradable hydrogel fertilizer	Water uptake 20 g g^{-1}; retained >500% of its weight in water after 10 days	>95% degradation within 60 days	Improved growth in a 24-d *Allium tuberosum* pot experiment	5
Lignin-derived cryogel (LC1)	Sustained relative mass retention under 35–75 °C and 50%	32.7% mass loss after 130 d	Increased germination and improved growth/physiological performance of maize, wheat, and rapeseed under greenhouse and field conditions	Present study

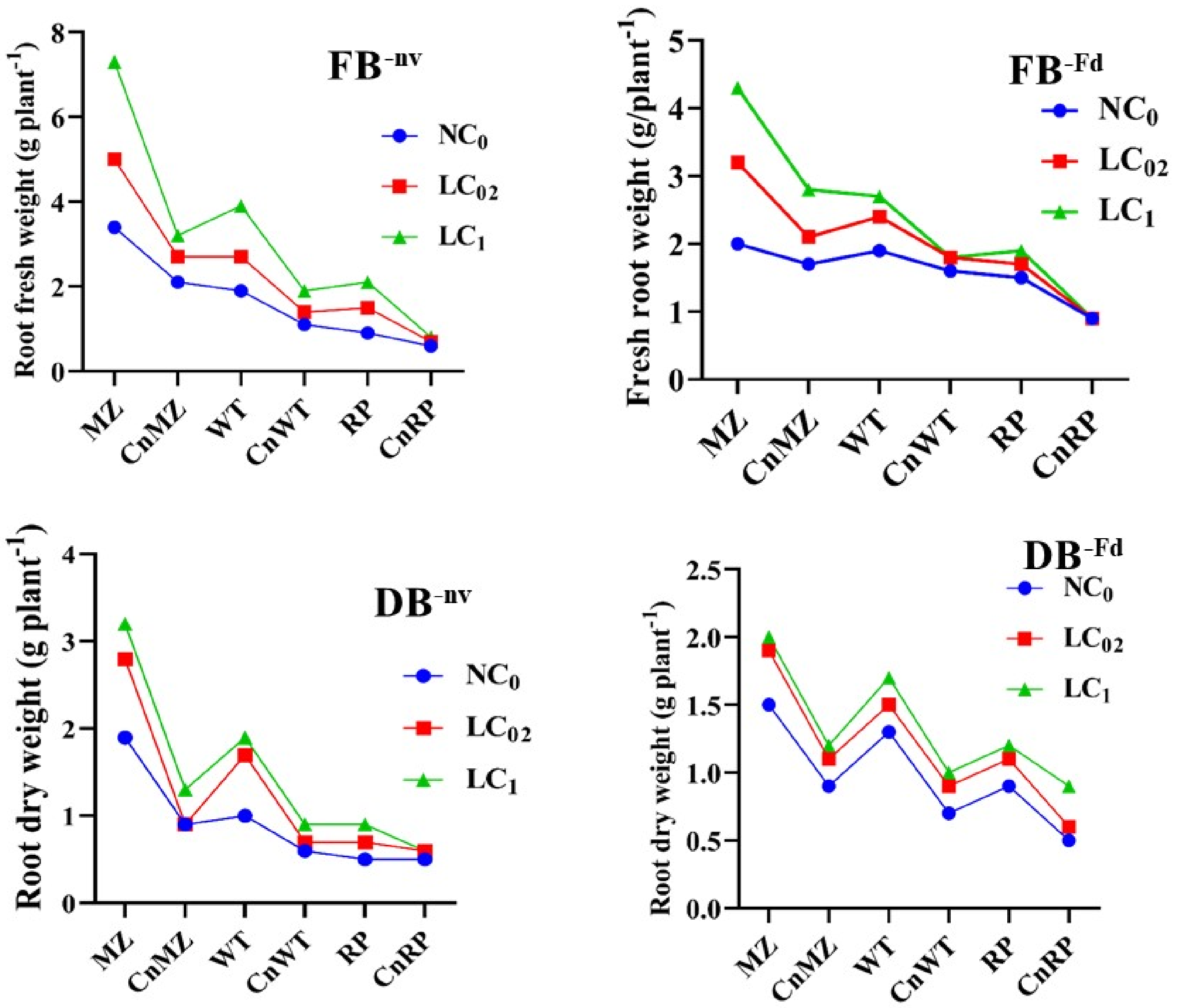

Fig. S1. Effect of lignin cryogel treatments on root elongation in selected crops under nv and Fd environmental conditions of weight by mass (g plants-).

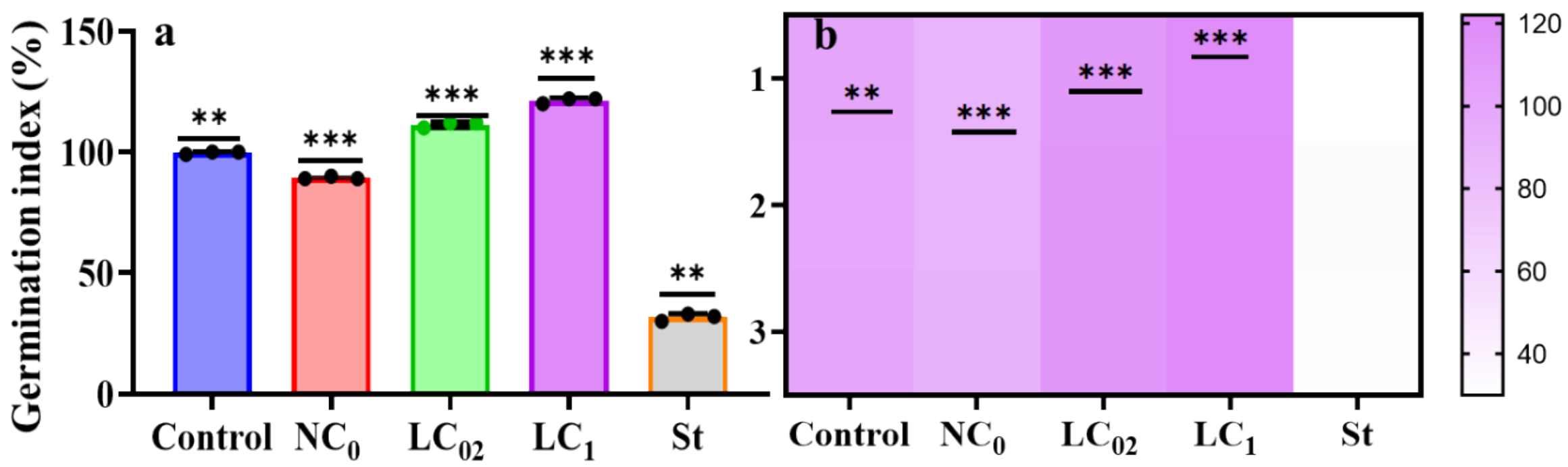

Fig. S2. Phytotoxicity and early seedling response to lignin-cryogel and synthetic hydrogel treatments. **(a)** Germination index (%) of seeds exposed to NC_0, LC_{02}, LC_1 and synthetic hydrogel (st) treatments relative to the untreated control demonstrating differential effects on germination

performance. Data are presented as mean ± standard deviation (n = 3). **(b)** Heatmap visualization of key physiological and morphological parameters, including root length, shoot length, fresh biomass, chlorophyll content, and relative water content, illustrating treatment-dependent variation in early seedling vigor. The color scale (white to purple) represents increasing magnitude of each parameter, with darker shades indicating stronger physiological performance. **Asterisks denote statistically significant differences compared with the untreated control (**$P < 0.01$, ***$P < 0.001$, Student's t-test).

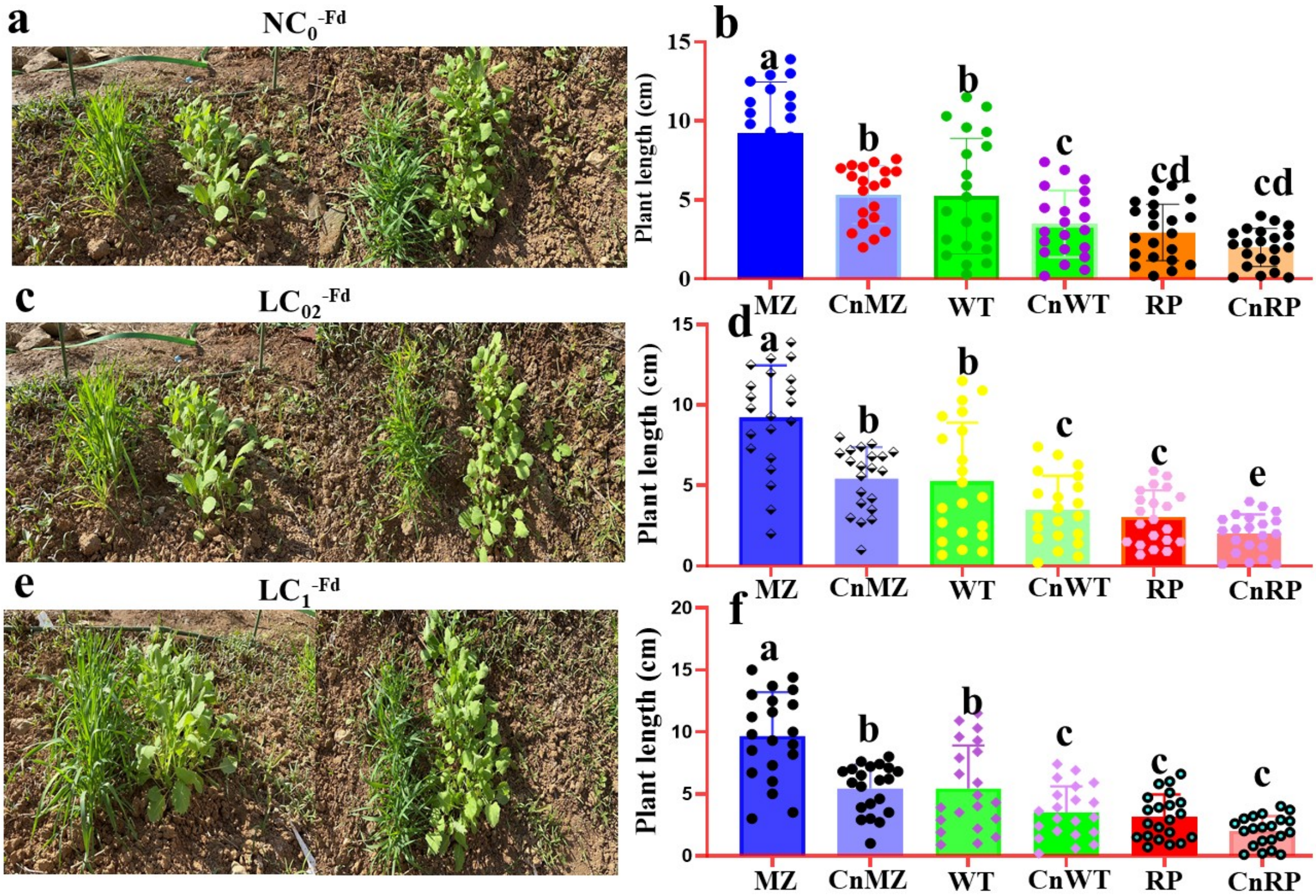

Fig. S3. Field validation of lignin-based formulations on early plant growth and canopy development under field (Fd) conditions across multiple crop species. **(a–c)** Representative field images illustrated plant growth following treatment with **(a)** NC₀⁻ᶠᵈ, **(b)** LC₀₂⁻ᶠᵈ, and **(c)** LC₁⁻ᶠᵈ cryogels. LC₁⁻ᶠᵈ treatments demonstrated visibly improved canopy density, plant vigor, and vertical growth compared with NC₀-Fd and LC₀₂-Fd treatments. **(d–f)** Quantitative analysis of plant height in MZ, WT, and RP under treatment with **(d)** NC₀⁻ᶠᵈ, **(e)** LC₀₂⁻ᶠᵈ, and **(f)** LC₁⁻ᶠᵈ cryogels. Bars indicate the mean plant height ± standard deviation, with individual biological replicates shown.

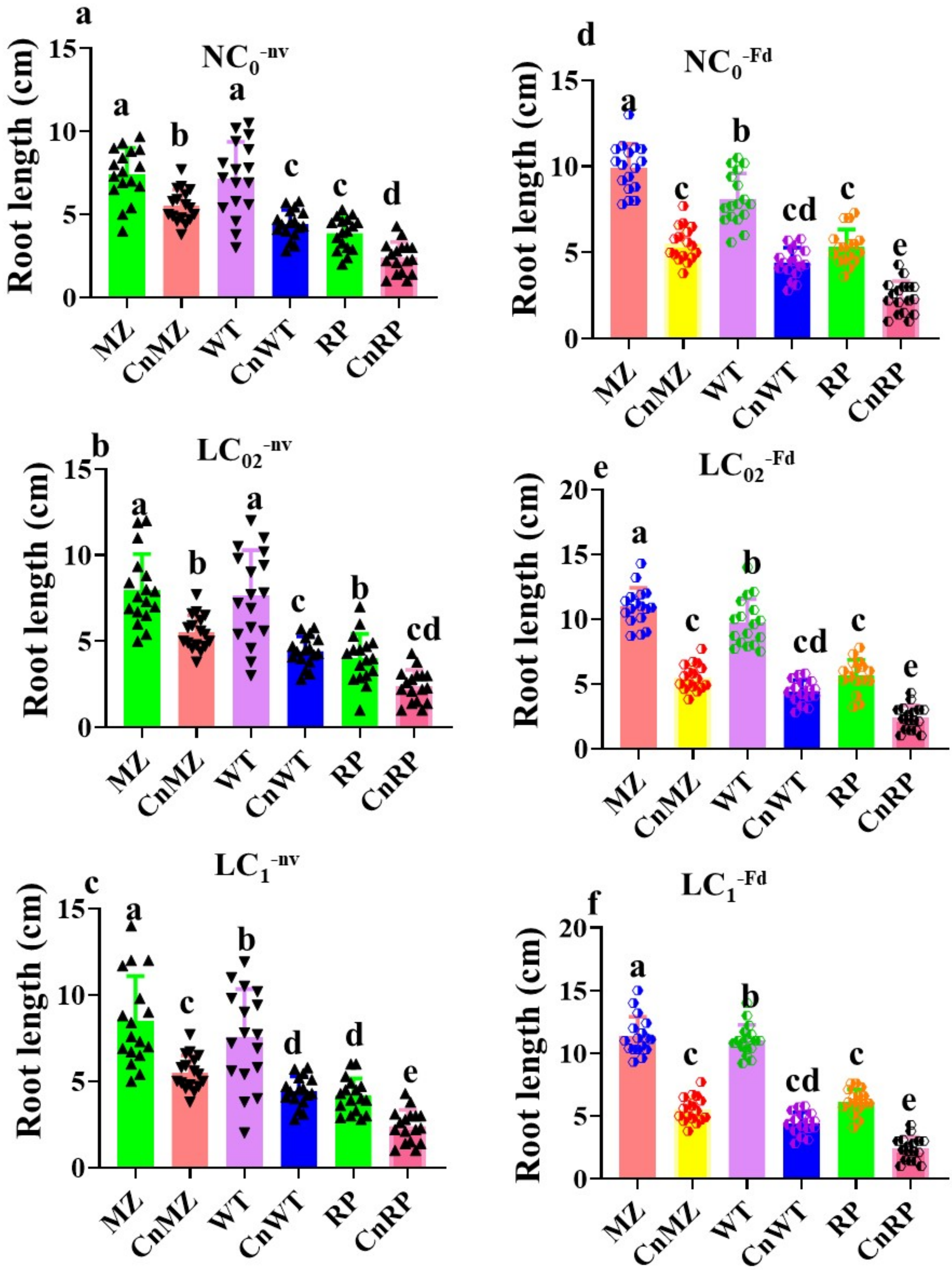

Fig. S4. Effect of lignin cryogel treatments on root elongation in selected crops under nv and Fd environmental conditions. **(a–c)** In vitro growth responses in NC_0–^{nv}, LC_{02}–^{nv}, and LC_1–^{nv}

treatments. **(d–f)** Field-derived conditions under NC_0–^{Fd}, LC_{02}–^{Fd}, and LC_1–^{Fd} treatments. Root dry and fresh weight (Fig. S1).

References

1. B. Song, H. Liang, R. Sun, P. Peng, Y. Jiang and D. J. I. J. o. B. M. She, International Journal of Biological Macromolecules, 2020, 144, 219-230.
2. T. A. Adjuik, S. E. Nokes, M. Montross, O. Wendroth and R. J. J. o. t. A. Walton, Journal of the ASABE, 2023, 66, 85-98.
3. B. Tomadoni, M. F. Salcedo, A. Y. Mansilla, C. Casalongué and V. A. J. E. p. j. Alvarez, European polymer journal, 2020, 137, 109953.
4. D. Das, N. Chingakham, M. Sarma, S. Basu and S. J. I. J. o. B. M. Bhaladhare, International Journal of Biological Macromolecules, 2025, 305, 141176.
5. S. L. Chiam, C. Leo and S.-Y. J. I. J. o. B. M. Pung, International Journal of Biological Macromolecules, 2025, 149056.
